# Comprehensive profiling and semi-quantification of exogenous chemicals in human urine using HRMS-based strategies

**DOI:** 10.1007/s00216-023-04998-9

**Published:** 2023-11-09

**Authors:** Daniel Gutiérrez-Martín, Esteban Restrepo-Montes, Oksana Golovko, Rebeca López-Serna, Reza Aalizadeh, Nikolaos S. Thomaidis, Montse Marquès, Pablo Gago-Ferrero, Rubén Gil-Solsona

**Affiliations:** 1 Department of Environmental Chemistry, Institute of Environmental Assessment and Water Research – Severo Ochoa Excellence Center (IDAEA), Spanish Council of Scientific Research (CSIC), 08034 Barcelona, Spain; 2 Institute of Sustainable Processes (ISP), Dr. Mergelina S/N, 47011 Valladolid, Spain; 3 Department of Analytical Chemistry, Faculty of Sciences, University of Valladolid, Paseo de Belén 7, 47011 Valladolid, Spain; 4 Department of Aquatic Sciences and Assessment, Swedish University of Agricultural Sciences (SLU), 75007 Uppsala, Sweden; 5 Laboratory of Analytical Chemistry, Department of Chemistry, National and Kapodistrian University of Athens, Panepistimiopolis Zografou, 15771 Athens, Greece; 6 Universitat Rovira I Virgili, Laboratory of Toxicology and Environmental Health, School of Medicine, IISPV, Sant LLorenç 21, 43201 Reus, Catalonia Spain; 7 Institut d′Investigació Sanitària Pere Virgili (IISPV), Reus, Spain

**Keywords:** Human biomonitoring (HBM), Non-target, Method validation, High-resolution mass spectrometry (HRMS), Deconjugation, Glucuronides

## Abstract

Chemicals infiltrate our daily experiences through multiple exposure pathways. Human biomonitoring (HBM) is routinely used to comprehensively understand these chemical interactions. Historically, HBM depended on targeted screening methods limited to a relatively small set of chemicals with triple quadrupole instruments typically. However, recent advances in high-resolution mass spectrometry (HRMS) have facilitated the use of broad-scope target, suspect, and non-target strategies, enhancing chemical exposome characterization within acceptable detection limits. Despite these advancements, establishing robust and efficient sample treatment protocols is still essential for trustworthy broad-range chemical analysis. This study sought to validate a methodology leveraging HRMS-based strategies for accurate profiling of exogenous chemicals and related metabolites in urine samples. We evaluated five extraction protocols, each encompassing various chemical classes, such as pharmaceuticals, plastic additives, personal care products, and pesticides, in terms of their extraction recoveries, linearity, matrix effect, sensitivity, and reproducibility. The most effective protocol was extensively validated and subsequently applied to 10 real human urine samples using wide-scope target analysis encompassing over 2000 chemicals. We successfully identified and semi-quantified a total of 36 chemicals using an ionization efficiency-based model, affirming the methodology’s robust performance. Notably, our results dismissed the need for a deconjugation step, a typically labor-intensive and time-consuming process.

**Graphical Abstract:**

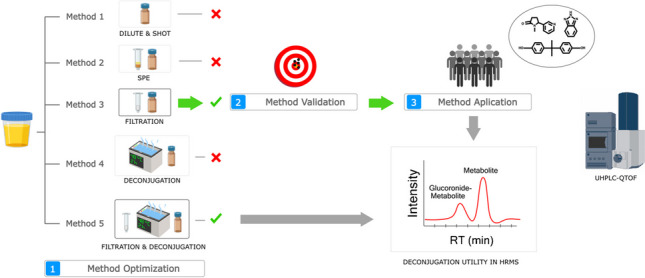

**Supplementary information:**

The online version contains supplementary material available at 10.1007/s00216-023-04998-9.

## Introduction

Contaminants of emerging concern (CECs) include a wide variety of pollutants such as endocrine-disrupting chemicals (EDCs), flame retardants, personal care products (PCPs), pharmaceutically active chemicals (PhACs), plasticizers, perfluorinated compounds (PFAS), pesticides, or their transformation products (TPs), among others [1]. Some of these chemicals may reach the human body from several exposure pathways, commonly by inhalation, ingestion, or dermal contact [2]. They may have adverse effects, even at low concentration levels, and prolonged exposure to these substances may result in severe and potentially fatal consequences [3]. To gain insight into the potential risks posed by CECs and establish effective regulatory measures, it is essential to have a comprehensive understanding of their presence and levels. Human biomonitoring (HBM) serves as the primary approach for investigating hazardous chemical exposures, enabling researchers to assess and analyze the presence of CECs in human samples [4].

Urine is one of the most used matrices in human biomonitoring studies for assessing exposure to various contaminants [5]. This preference stems from its high sample volume availability, ease of collection, and non-invasive nature compared to other biofluids such as blood [6]. Hence, numerous studies have focused on urine to screen the presence of CECs, often employing target strategies with low-resolution mass spectrometers (LRMS) as triple quadrupoles (QqQ). Conventionally, investigations of CECs in urine have primarily concentrated on the analysis of specific chemical families with low-resolution mass spectrometers, including EDCs [7–9], flame retardants and plasticizers [10–13], PCPs [14, 15], UV filters [16, 17], doping agents [18], PhACs and drugs of abuse [19–21], PFAS [22–24], or biocides [25–27]. Recently, there has been a notable increase in studies that aim to achieve the simultaneous analysis of environmental pollutants from multiple chemical families, as demonstrated by Lee et al. [28], with their examination of 86 chemicals. However, while LRMS target screening strategies have proven to be robust, reliable, and sensitive, they are inherently limited in terms of the number of chemicals that can be screened. This limitation arises from the requirement of preselecting contaminants and relying on QqQ instruments that acquire information solely through ion transitions. Consequently, this approach introduces potential bias in the study design and can lead to the occurrence of the Matthew effect, as discussed by Daughton [29].

Recent advances in high-resolution mass spectrometry (HRMS) have opened new possibilities for the application of wide-scope target screening and suspect and non-target strategies in HBM, leading to a broader characterization of the chemical exposures (e.g., [30, 31]). However, a key limitation of these methods is the requirement of non-selective sample treatments, since they aim to simultaneously analyze a wide range of chemicals with varying physicochemical properties. Thus, a compromise between universality and sensitivity is needed, being accentuated in samples where a high matrix effect is expected, such as urine [32]. In contrast, these advanced techniques provide noteworthy benefits. Firstly, they eliminate the requirement for preselecting target chemicals, allowing for a holistic characterization of CECs in a single analysis [33]. The primary limitation lies in the current state of knowledge (e.g., databases) and the slightly low sensitivity of HRMS instruments compared with QqQ [34]. Secondly, they offer the possibility to retrospectively search for specific information in previously acquired datasets [35]. Finally, these techniques enable the identification of metabolites, particularly valuable in urine samples where certain chemicals may undergo phase I (e.g., oxidation) or phase II (e.g., bind to glucuronide, sulfates, or glutathione moieties) metabolism [36].

Classic QqQ-based target strategies fail to detect these metabolites and a common strategy to address this challenge involves performing deconjugation steps to hydrolyze phase II metabolites and recovering the unmetabolized chemicals, which is an essential step in these studies [32]. Although this step simplifies data processing and enables accurate quantification of all CECs, it introduces complexities and increases costs [37]. Moreover, the incubation of samples at relatively high temperatures for extended periods of time during deconjugation may potentially degrade specific compounds.

The current trend in HRMS studies aims to avoid deconjugation steps by relying on the comprehensive information provided by full-scan data to elucidate metabolites. However, when deconjugation is not applied, the quantification of CECs needs to include the corresponding phase I and II metabolites. Hence, (semi)quantitative results can be usually retrieved from HRMS-based data through suspect and non-target analysis. In this case, one way to derive quantitative data is to consider the exerted ratio—if known—between parent compounds and transformation products. Therefore, the calibration curve of the parent compound can be used for quantitative analysis by applying the correction factors [38, 39]. Another popular method for HRMS-based semi-quantitative analysis is to use similar chemical structures. The calibration curves and reference standards are usually available for similar pairs, and they can be used for compounds whose reference standards are not available. Apart from the unknown and diverse ionization behavior of similar pairs, these methodologies assume that the matrix effect and the recovery of the analytical method are minimal and satisfactory, respectively. In addition, the ionization efficiency (IE) of analytes needs to be considered while performing semi-quantification analysis to avoid misinterpretation of low abundant analytes showing high IE [40, 41]. These approaches enable semi-quantitative non-target strategies without the need for laborious deglucuronidation steps.

The identification and (semi)quantification of CECs in urine samples using LC-HRMS require validated methodologies to analyze a diverse range of chemicals. Such methodologies are essential for evaluating the risks these CECs pose to human health and are currently in high need. To the best of the authors’ knowledge, there are only a few examples of this kind of methodology application [36, 37, 42]. The primary goal of the present study was to validate and implement an analytical methodology utilizing LC-HRMS for the analysis of a wide range of CECs in urine samples. Hence, five different extraction methodologies were evaluated in terms of trueness, sensitivity, and matrix effect. Subsequently, the one with the best performance was further validated using a set of 90 chemicals with a wide range of physicochemical properties and applied to analyze 10 real urine samples. Additionally, the necessity of a deconjugation procedure to account for glucuronidated chemicals in HRMS-based protocols was assessed using real samples.

## Materials and methods

### Reagents and materials

Methanol (MeOH) (HPLC-grade), water (HPLC-grade), formic acid (> 99% purity), ammonium acetate, and ammonium formate (≥ 99.0% purity) were purchased from Merck (Darmstadt, Germany). Distilled water was obtained by a Milli-Q purification system (Aurium, PRO-VFT, Sartorius, Göttingen, Germany). Ammonia solution (32%) was purchased from VWR Chemicals (France).

For the *Glu* and *Cap-Glu* protocols (described in “Sample preparation optimization”), enzyme β-Glucuronidase from *E. coli* K12 was obtained from Merck (Darmstadt, Germany), and Captiva 3 mL Non-Drip filter cartridges were bought from Agilent (Madrid, Spain). For the SPE protocol (described in “Sample preparation optimization”), solid-phase extraction (SPE) empty cartridges, frits, Sepra ZT (30 µm, 85 Å) powder, Sepra ZTL-WCX (100 µm, 300 Å) powder, and Sepra ZTL-WAX (115 µm, 330 Å) powder were purchased from Phenomenex (Madrid, Spain), and Isolute ENV + was purchased from Biotage (Uppsala, Sweeden).

Analytical standards, including the isotopically labelled internal standards (IS), were purchased from Merck (Darmstadt, Germany) and LGC Standards (Barcelona, Spain). A mix of labelled ISs at 1 mg·L^−1^ was prepared by mixing appropriate aliquots of each standard stock solution in MeOH. Similarly, a mix of standards at 1 mg·L^−1^ was also prepared (SI-1, Table S1). Further information regarding the analytical standards is provided in previously published literature [43]. ChatGPT-3.5, an OpenAI tool, was used only to refine the English language.

### Sample collection

Human urine samples were collected from 15 volunteers, using a prospective and randomized study conducted in Tarragona County (Catalonia, Spain) between the 1st and the 5th of March of 2021. We used first-morning void (FMV) in sterile polypropylene urine collection vessels. Regarding demographic characteristics, we obtained samples from 7 males and 8 females, in the age range from 25 to 45 years. We pooled 5 of these samples for validation purposes, and individual samples from the other 10 volunteers were used for real sample analysis. The study was approved by the Ethics Committee concerning Research into People, Society and the Environment of the Universitat Rovira i Virgili (CEIPSA- URV; Ref: CEIPSA-2020-PR-0003).

### Sample preparation optimization

Five sample treatment protocols were applied to urine by using the composite urine pooled sample. The protocols, named *Centrifugation (Cent)*, *Solid phase extraction (SPE)*, *Deconjugation (Glu)*, *Captiva filtration (Cap)*, and *Captiva filtration followed by deconjugation (Cap-Glu)*, are described below.

After adding a labelled internal standard as a surrogate (clothianidin-d_3_ at 50 µg·L^−1^ calculated in the final extract), a centrifugation step (3500 rpm, 5 min) of 1 mL of pooled sample (2 mL in SPE protocol) was performed in all the protocols. Then:

***• Cent***: the supernatant (475 µL) was collected and diluted with methanol until 500 µL in a chromatographic vial.
***• SPE***: the supernatant (1.5 mL) was transferred to a previously baked glass bottle, where it was diluted up to 10 mL in acidified water (pH=6.5, formic acid/ammonia). A solid-phase extraction was performed with mixed-mode homemade cartridges. Details about the SPE protocol can be found elsewhere [33]. The extract was evaporated until dryness and reconstituted with H_2_O:MeOH (1:1) to a final volume of 150 µL.
***• Glu***: the supernatant (500 µL) was mixed with 1 mL of buffer (ammonium formate, 1 M) at pH=6.2 (adjusted with ammonia and formic acid) and 25 µL of the enzyme (β-glucuronidase). Deconjugation was performed at 48 °C for 2 h as suggested by previously published literature [44, 45]. Finally, 950 µL of the resulting solution was mixed with 50 µL of methanol in a chromatographic vial.
***• Cap***: the supernatant was passed through a Captiva filtration cartridge, and 475 µL of the filtrated solution was mixed with 25 µL of methanol in a chromatographic vial. This protocol has been adapted from elsewhere [36].
***• Cap-Glu***: the supernatant was passed through a Captiva cartridge. Then, 500 µL of the resulting extract followed the *Glu* protocol (described above).

All the chromatographic vials were stored frozen (− 80 °C). Just before the LC-HRMS analysis, all vials were spiked with the IS mixture to a 50 ng·mL^−1^ in-vial concentration.

### UHPLC-QTOF: instrumentation and conditions

An ultra-high performance liquid chromatography (UHPLC) system with a Bruker Elute Pump HPG 1300 coupled to a QTOF Impact II (Bruker, Bremen, Germany) was employed for the analysis. The chromatographic separation was done with a Bruker Intensity Solo HPLC Column (C18–2, 1.8 µm, 2.1 × 100 mm) preceded by a guard column (CORTECTS C18, 1.7 μm 2.1 × 5 mm, Waters, Milford, USA) both maintained at 40 °C during analysis. The mobile phase in positive ionization mode (+ ESI) consisted of water/methanol (99:1) with 5 mM ammonium formate and 0.01% formic acid (aqueous phase—A) and methanol with 5 mM ammonium formate and 0.01% formic acid (organic phase—B). In negative ionization mode (-ESI), water/methanol (99:1) with 5 mM ammonium acetate (A) and methanol with 5 mM ammonium acetate (B). The mobile phase gradient for both + ESI and -ESI is summarized in supplementary information (SI-2, Table S2).

The operating parameters of the electrospray ionization interface (ESI) were similar for both polarities: end plate offset 500 V, capillary voltage 2500 V, nebulizer 3 bar, dry gas 8.0 L·min^−1^, dry temperature 200 °C, probe gas temperature 200 °C, and probe gas 4.0 L·min^−1^. The injection volume was 10 µL.

The QTOF system operates in broadband collision-induced dissociation (*bbCID*), a data-independent acquisition (DIA) mode, where two sequential full-scan events are performed. In + ESI, the first scan applied a low collision energy (6 eV) generating a low-energy full-scan function while the second one applied a high collision energy ramp (stepped from 24 to 36 eV) resulting in a scan containing the fragmentation information of all ions present there. Scans were performed in the range *m/z*: 70–1000 Da. In -ESI, the first scan applied low collision energy (8 eV) while the second one applied a high collision energy ramp (stepped from 24 to 36 eV). Scans were performed in the range *m/z*: 70–1000 Da. The scan rate was 3 scans per second. These chromatographic and mass spectrometer conditions were applied to all the injections.

Post-acquisition data treatment was performed with Compass *DataAnalysis 5.0* and *TASQ 2.1* software (Bruker Daltonics, Bremen, Germany).

### Selection of sample preparation method and method validation

The method validation was performed in two stages. In the initial stage, the five proposed methodologies were assessed in terms of recovery, trueness, sensitivity, and matrix effects to discern their comparative performances. Subsequently, the most promising technique was selected for comprehensive validation. The validation was conducted using a list of 90 chemicals, chosen to ensure the coverage of a wide range of physicochemical properties (including logKow, chemical class, and heteroatoms in the structure), so the use of the method may be extrapolated to a wider range of chemicals in a non-target manner. Finally, a wide-scope target screening was applied to 10 human urine samples using the validated extraction methodology. Additionally, this set of samples underwent a treatment methodology that included a deconjugation step. This was performed to assess its significance in HRMS-based protocols when using real samples.

#### Comparison of the different sample treatments

To determine the most effective sample treatment method among the five evaluated, we conducted subsequent experiments and calculations. Fifteen urine replicates were spiked at a concentration of 10 µg·L^−1^ (calculated in the final extract) with a standard mixture (SI-1, Table S1) before centrifugation (*pre-spiked samples*). Additionally, twenty replicates were prepared without the addition of any standard, serving as *non-spiked samples*. Each of the five treatments described in “Sample preparation optimization” was applied to three *pre-spiked samples* and to four *non-spiked samples*, resulting in a total of seven samples per treatment. Within each treatment, three out of the four *non-spiked* samples were spiked with the standards mixture at the end of each protocol (referred to as *post-spiked samples*) to evaluate the recovery of each sample treatment, while the remaining non-spiked samples were used as a protocol blank. To assess the matrix effect, a compound mixture in a solvent (MeOH:H_2_O, 5:95, *v/v*) was also injected. To account for the chemicals already present in the pooled urine sample, the peak area from the protocol blank was subtracted from both pre-spiked and post-spiked samples.

The trueness was evaluated with the recoveries (*R*%) of the extraction, determined by dividing the average *pre-spiked* (*n* = 3) by the average (*n* = 3) *post-spiked* peak area for each analyte:

1 $$\mathrm R\%=\frac{\mathrm{Area}\;\mathrm{for}\;\mathrm{each}\;\mathrm{analyte}\;\mathrm{in}\;\mathrm{pre}\;-\;\mathrm{spiked}\;\mathrm{sample}}{\mathrm{Area}\;\mathrm{for}\;\mathrm{each}\;\mathrm{analyte}\;\mathrm{in}\;\mathrm{post}\;-\;\mathrm{spiked}\;\mathrm{sample}}\cdot100(\%)$$

The sensitivity was assessed with instrumental limits of detection (LODs), estimated as the concentration which corresponds to an *S/N* ratio of 3, as well as instrumental limits of quantification (LOQs), with the Eqs. 2 and 3:

2 $$\mathrm{LOD}=3\cdot\frac{\mathrm{Concentration}\;\mathrm{of}\;\mathrm{the}\;\mathrm{analyte}}{\frac SN\mathrm{ratio}\;\mathrm{for}\;\mathrm{the}\;\mathrm{analyte}\;\mathrm{at}\;\mathrm{the}\;\mathrm{given}\;\mathrm{concentration}}(\mathrm{ng}\cdot\mathrm{mL}^{-1})$$

3 $$\mathrm{LOQ}=\frac{10}{3}\cdot \mathrm{LOD }(\mathrm{ng}\cdot {\mathrm{mL}}^{-1})$$

The matrix effect (ME) was evaluated by dividing the average peak area for *post-spiked samples* (*n* = 3) with the peak area obtained from spikes in the solvent for each analyte.

4 $$\mathrm{ME\%}=\frac{\mathrm{Area\,for\,each\,analyte\,in\,postspiked\,sample}}{\mathrm{Area\,for\,each\,analyte\,in\,solvent}}\cdot 100(\mathrm{\%})$$

#### Method validation parameters

Based on the previous results, the best methodology was further validated to demonstrate the suitability of the analytical method for the analysis of urine by LC-HRMS in terms of extraction recoveries, matrix effect, sensitivity, linearity, linear range, and inter- and intra-day precision.

Quintuplicate urine aliquots were prepared by spiking with standard chemicals (SI-1, Table S1) at three different fortification levels (2, 10, and 50 µg·L^−1^ in the final extract) prior to sample treatment. Additionally, a quintuplicate was prepared without the addition of any standard (*non-spiked* sample). A matrix-matched calibration curve consisting of 9 concentration levels (0, 0.05, 0.2, 0.5, 1, 5, 10, 50, 100 µg·L^−1^) was prepared by adding standard chemicals to a *non-spiked* sample. This calibration curve was used to evaluate the linearity of the method and calculate the recoveries. Additionally, non-spiked samples were used as blanks to account for any possible sample interferences or pre-existing chemicals in the pooled urine.

The trueness of the method was assessed using the recoveries (*R*%), which were calculated in a similar manner to the method performance step (see Eq. 1), but using the calculated concentration obtained from the matrix-matched calibration curve and the theoretical concentration instead of the peak areas. The limits of quantification (LOQs) were determined as the lowest concentration where a peak was observed on the matrix-matched calibration curve. The LODs were calculated as 3/10 of the LOQ values. The linear range was set between the LOQ and the highest concentration on the calibration curve while maintaining linearity. The matrix effect (ME) was estimated in the same manner as during the pre-validation step, following Eq. 4. Precision was evaluated by calculation of the coefficient of variation (CV%) of the peak area from a quality control sample (10 µg·L^−1^) injected several times. This quality control sample consisted of the pooled mixture spiked with standards (10 µg·L^−1^) and IS (50 µg·L^−1^) and was then processed identically to all other samples. Concretely, intra-day precision (also called repeatability or intra-day reproducibility), was calculated by injecting the quality control sample 9 times within the same day, while intermediate precision (also called inter-day reproducibility) was determined by injecting the quality control sample 9 times over three different days.

#### Applicability of the method

Finally, the validated methodology was employed to analyze 10 human urine samples, evaluating the performance of the methodology in wide-scope target and suspect screening and also evaluating the usefulness of deconjugation steps in sample preparation for HRMS analysis by performing both protocols (*Cap* and *Cap-Glu*). HRMS-acquired data was processed by matching compounds included in a large in-house produced database (> 2000 chemicals). To ensure accurate results and minimize false positives, each compound in all samples underwent manual inspection, aided by criteria such as the accurate mass score, the retention time (RT) score, and the isotopic fit score. Finally, the chemicals were semi-quantified using a model for ionization efficiency (IE) based on the quantitative structure-ionization relationship model (QSIR). The details of QSIR model development and validation workflow can be found elsewhere [40]. Here, the IE database is developed based on the logarithmic ratio between the slope (from calibration curves) of the analyte of interest and a reference compound (-ESI: bisphenol G and + ESI: O-desmethyl venlafaxine). This way, the logIE is normalized relatively to the baseline IE value to reflect the true ionization range (low and high ionization efficiency scale). Furthermore, the ionization efficiency models (for negative and positive electrospray ionization modes) were projected to the urine matrix through few representative exogenous chemicals called calibrants to account for IE changes due to the urine matrix and background ions. In case of poor correlation, matrix-specific model was developed using matrix-matched calibration curves. Here, the main adduct forms of [M-H]^−^ and [M + H]^+^ are used for calculating peak area and log_2_IE values. The list of compounds and the details of QSIR models as well as logIE database for urine samples are available in SI-3.

### Quality control and quality assurance (QC/QA)

Quality control and quality assurance (QC/QA) measures were applied to avoid potential contamination during the sample treatments or instrumental analysis. Hence, all glass material was cleaned (Milli-Q water and acetone) and heated (450 °C, 6 h) before use, and the working bench was cleaned with water and acetone before a clean material was placed.

The performance of the instrument was also controlled by the following measures. Quality controls (consisting of a urine sample spiked at 10 µg·L^−1^) were injected every 20 injections to evaluate the instrumental performance. Procedural blanks (3 per 10 real samples) were carried out (using Milli-Q water instead of urine) for each extraction protocol. The average peak area of the blanks plus 3 times the standard deviation was subtracted from the peak area of the 10 urine samples. Methanol was injected every 10 injections to control the carryover. Additionally, all samples were spiked with a surrogate internal standard to evaluate any compound loss during the sample treatment (clothianidin-d3), and the rest of ISs (*n* = 20) were spiked just before the instrumental analysis to control for possible signal reduction during the LC-HRMS analysis. The HRMS instrument was externally calibrated before sequence using a sodium formate solution before each analytical batch. Additionally, in the first 15 s of each acquisition, sodium formate infusion was acquired, to perform an internal calibration for each injection.

## Results and discussion

For the method validation, a carefully curated set of 90 chemicals was chosen to ensure the coverage of a wide range of physicochemical properties. These chemicals were selected based on their ability to elute at diverse chromatographic RT and were distributed evenly between positive and negative ionization modes (IM) during instrumental acquisition. Specifically, 39 chemicals ionized in the positive electrospray ionization (+ ESI) mode, and 42 chemicals in the negative (-ESI) mode. Furthermore, 9 chemicals exhibited ionization capability in both modes. The selected chemicals demonstrated a range of logP values, with values ranging from − 0.2 to 7.4 for + ESI and from − 0.1 to 6.4 for -ESI. To ensure comprehensive coverage, a specific range of retention times was also considered, with a selected range of 3 to 14 min for + ESI and 4 to 12 min for -ESI. The list of chemicals included a diverse range of compound classes such as pharmaceuticals, biocides, industrial chemicals (including UV filters and PCPs), flame retardants, plasticizers, per- and polyfluoroalkyl substances (PFASs), and food and tobacco-related chemicals. Additionally, transformation products were included, resulting in a wide variety of chemical structures. A summary of the selected chemicals can be found in Table S1 (SI-1), and Figure S.2. (SI-4) provides a visual representation of the chemical diversity within the set.

### Method selection

**Fig. 1:**
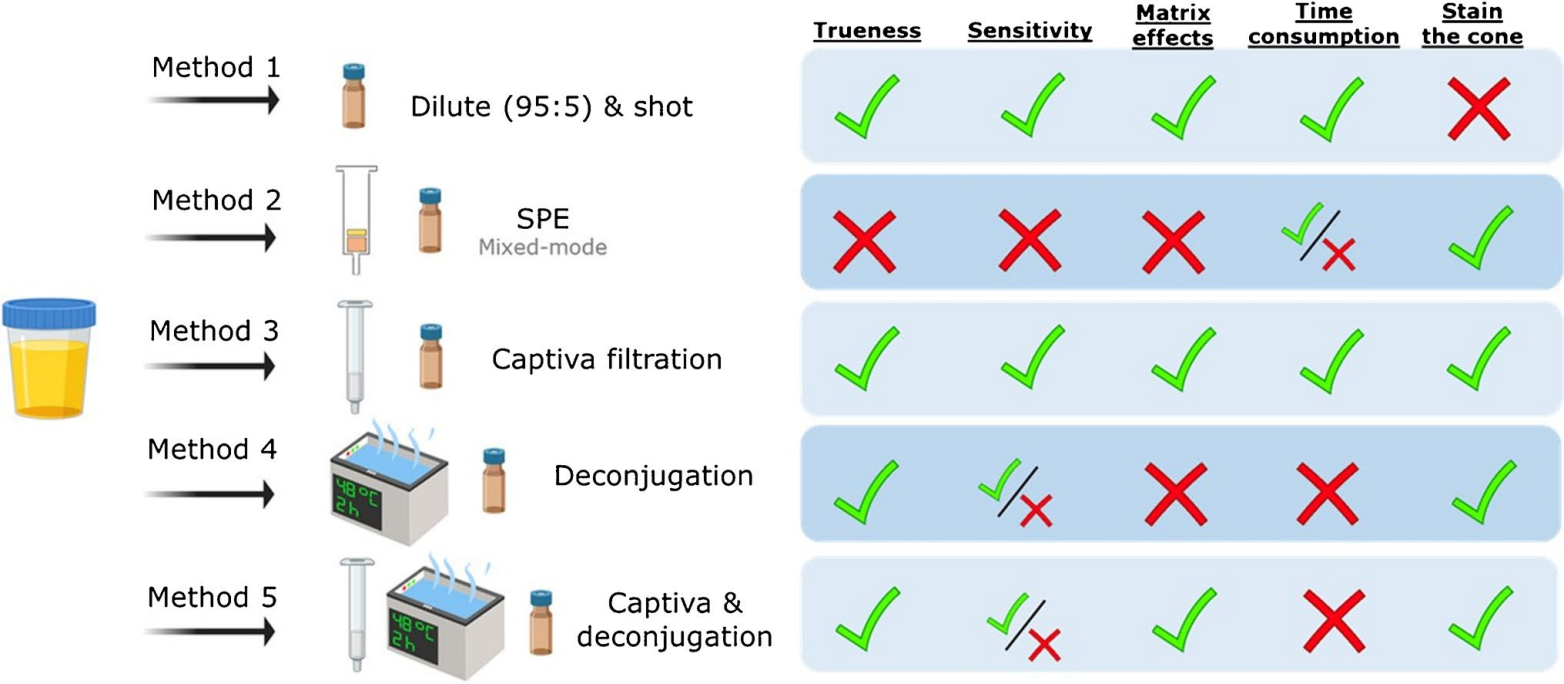
Method selection overview

The five methodologies were evaluated in terms of trueness, sensitivity, and matrix effects, and the findings are summarized in SI-5, Table S4 and illustrated in Fig. 1. Generally, the methodologies *Cap* and *Cent* showed the highest recoveries, with approximately 60% of the chemicals showing recoveries between 70 and 110%. On the other hand, the *Glu* and *Cap-Glu* methods exhibited lower recoveries, with around 85% of the chemicals failing within the 30% and 70% recovery range. This discrepancy could be attributed to the deconjugation step, as the extracts underwent significant heating (48 °C) for 2 h, which may have resulted in degradation. Despite more conservative protocols could have been deemed (e.g., lower temperature, in the range of 30 °C, with higher reaction time), the analytical methodology was designed for large sample cohorts. Therefore, time efficiency, as exemplified by protocols such as *Cap*, was prioritized over *Glu* or *Cap-Glu*, particularly when handling a significant number of samples, such as over 1000.

In addition, the inclusion of deconjugation steps in the *Glu* and *Cap-Glu* methodologies necessitated sample dilution, potentially compromising the detectability of chemicals at low concentrations, especially when assessing the human exposome. Moreover, deconjugation protocols involved the addition of salts, which could potentially affect ionization efficiency, particularly when using high injection volumes, despite the use of relatively volatile salts such as ammonium formate. Moreover, considering that deconjugation was not observed to be crucial for HRMS-based analysis, as the overall results did not show a significant advantage compared to the *Cap* protocol, both the *Glu* and *Cap-Glu* protocols were discarded from further analysis.

The primary objective of the solid-phase extraction (*SPE*) method was to assess the impact of a preconcentration and clean-up step on method sensitivity. However, almost all detected chemicals presented ME% in the range of 0–40% (being 0% total suppression) when using the *SPE* method. This indicated that the preconcentration step negatively affected the LODs for these chemicals. Despite the expectation that the analytes of interest would be retained in the *SPE* cartridge, the high complexity of the matrix led to poor results. This was also observed in the surrogate signal, which showed an area that was an order of magnitude lower compared to other protocols. Therefore, the *SPE* method was also discarded for further analysis.

Finally, both *Cent* and *Cap* presented quite similar results in terms of LODs. However, the *Cent* methodology presented an additional problem, namely a loading effect observed on the mass spectrometer cone during the instrumental analysis. Although this issue did not directly affect the estimation of *R*%, ME%, and LODs during the short validation batch, it had the potential to cause severe hardware problems in larger batches. Such complications could lead to a drastic reduction in signal intensity and make the method unsuitable for unattended analysis. Thus, considering the necessity for a methodology capable of analyzing large cohorts, the *Cent* method was also discarded. Ultimately, the *Cap* methodology was selected as the final protocol to undergo a complete validation.

### Instrumental parameter optimization

Instrumental parameters, such as injection volume, chromatographic gradient, or source conditions, are critical to obtain the best sensitivity and robustness in these analytical methodologies. The chromatographic gradient was adapted from [43]. Both the TargetScreener HR 4.0 database (including > 2800 compounds) and the in-house-developed database (including > 2000 chemicals) have been acquired under this same gradient. Consequently, chromatography parameters were not further optimized, as RT matches required the use of the same exact conditions. Source parameters were also adapted from the TargetScreener HR 4.0 manual. The resulting methodology must deal with a wide range of chemical properties, and those conditions were specifically optimized for the instrument source.

The injection volume is a critical parameter to consider when optimizing LC–MS methods. While higher injection volumes are typically expected to improve sensitivity, they can also lead to decreased sensitivity if heavy matrix effects are present, especially in complex matrices such as urine. In order to optimize the injection volume, a test was conducted using different volumes (2, 5, and 10 µL). Urine samples treated with the *Cap* protocol were spiked at two different concentration levels (0.5 and 10 µg·L^−1^). Higher injection volumes were not considered to avoid potential issues such as column overload, compromised peak shape, and mass spectrometer cone load effect, as well as to prolong the chromatographic column lifespan. Results are here summarized and further illustrated in SI-6, Figure S.3. The best LODs were achieved with a 10 µL injection volume, especially at 0.5 µg L^−1^ concentration level. This suggests that any increase in matrix effects, if present, was offset by the signal gain obtained through higher analyte input at the larger injection volume. Additionally, the number of chemicals with a signal higher than 10^4^ at 0.5 µg·L^−1^ fortification level increased from 46%, injecting 2 µL, to 50% and 53% injecting 5 and 10 µL, respectively, indicating an improvement in method sensitivity. Similar trends were observed at the 10 µg·L^−1^ concentration level, with percentages increasing from 78 to 85% and 94%, respectively. Additionally, 72% of the spiked chemicals were detected at 0.5 µg·L^−1^, providing sufficient method sensitivity to accomplish its analytical purpose. Hence, an injection volume of 10 µL was selected as the optimal choice.

### Method performance

**Fig. 2:**
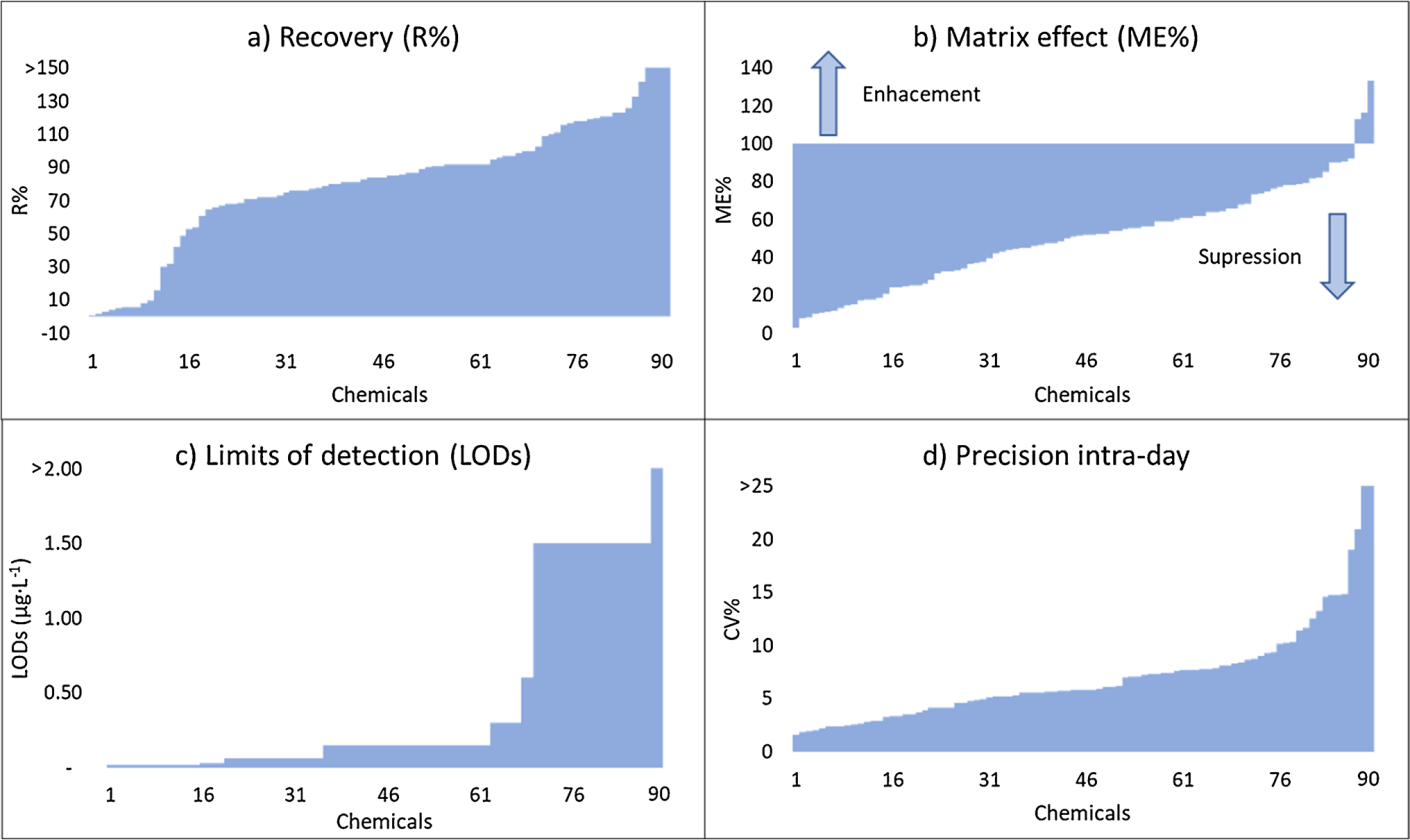
Method validation results for Cap protocol. Refer to SI.7, Table S5, for detailed data. **a** Recoveries (10 µg·L^−1^ fortification level), **b** matrix effects, **c** limits of detection, and **d** precision intra-day

The *Cap* methodology was ultimately chosen for full validation, building upon its successful performance in previous works [36] involving 8 labelled internal standards. However, the present study was more ambitious as it aimed to evaluate its performance on a larger number of analytes, encompassing 90 standards. The main goal was to investigate the suitability of the methodology for suspect and non-target analysis, along with the potential for further (semi)quantification of the identified chemicals. A comprehensive summary of the full validation results is summarized in Fig. 2 and SI-7, Table S5.

The method demonstrated good sensitivity, with LOQ in the range of 0.05 to 10 µg·L^−1^. Indeed, 67 compounds (which represent 74% of the chemicals assessed) showed LOQs ≤ 1 µg·L^−1^. This is of utmost importance for HBM studies as exogenous chemicals are usually present at low concentrations (usually in the range of ng·L^−1^ to µg·L^−1^). Thus, method sensitivity is sufficient to detect CECs in real human urine samples [36]. The recoveries were overall satisfactory, with approximately 60% of the chemicals in the range of 70–120%, at the three fortification levels. Only 9 chemicals showed a poor recovery rate below 10%. Despite some limitations, the *Cap* method displayed good sensitivity and recovery for the vast majority of the spiked compounds.

The matrix effect observed in the *Cap* method resulted mostly in signal suppression, as previously observed during the pre-validation test. This was attributed to the high complexity of the matrix. However, it is worth noting that only 3 chemicals presented a suppression exceeding one order of magnitude when compared to the signal in the solvent. This finding demonstrates the effective clean-up efficacy of the Captiva cartridges used in the method.

Additionally, the regression coefficient (*R*^2^) for the matrix calibration curve was > 0.99 for 96% of the chemicals, being always higher than 0.98. Regarding the dynamic range, linearity was observed in the range of LOQ–100 µg·L^−1^ for the majority of the chemicals. Only five chemicals showed linearity extending up to 50 µg·L^−1^, which was still deemed acceptable considering that concentrations higher than 100 µg·L^−1^ were not typically common in human urine.

Finally, intra-day reproducibility, expressed as CV%, was found to be in the range of 2–20% for 87 chemicals, and lower than 10% for 75 chemicals. Inter-day reproducibility showed a higher variability, but with satisfactory results for 70 chemicals (CV < 20%) and only 3 showing CV > 30%.

The comprehensive evaluation of the *Cap* method demonstrated that all tested parameters met the validation criteria, affirming its suitability for suspect and non-target analysis, including (semi)quantitation. These results provide a thorough analytical quality overview, ensuring the method’s performance and reliability for its intended purpose.

### Deconjugation in HRMS-based strategies

To test the capabilities offered by HRMS-based strategies in eliminating the need for deconjugation steps, the validated *Cap* protocol and *Cap-Glu* protocol were employed to analyze 10 urine samples retrieved from ten volunteers. The goal was to assess the feasibility of utilizing semi-quantitative analysis for both contaminants and their glucuronide metabolites, thereby avoiding the deconjugation process. To ensure results comparability, the *Cap-Glu* methodology was also subjected to validation and yielded acceptable results (SI-8, Table S6).

**Fig. 3:**
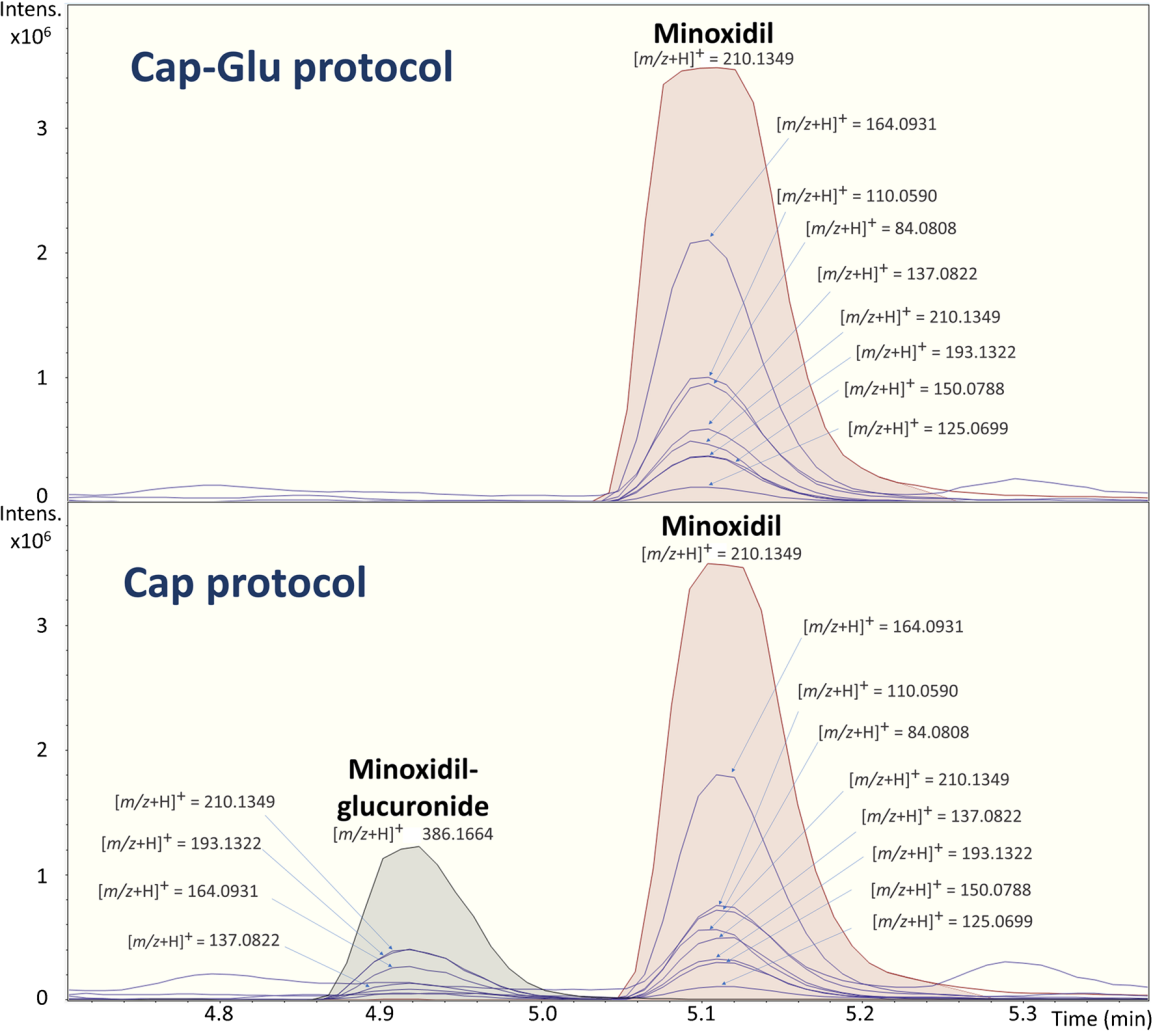
Minoxidil (RT: 5.1 min) and minoxidil glucuronide (RT: 4.9 min) chromatographic peaks for both *Cap* and *Cap-Glu* protocol. Main fragments are included

After applying a wide-scope target screening, we searched for tentative glucuronide metabolites using a suspect screening approach. We identified chemicals that exhibited a *m/z* increase of 176.0317, corresponding to the addition of C_6_H_10_O_7_ to the molecular formula and loss of H_2_O. These chemicals demonstrated to undergone phase II metabolism, leading to the formation of their glucuronide conjugates. The glucuronide metabolites presented lower RT, due to their increased polarity compared to the free form of the chemical. Also, they shared some fragment ions with the parent compound. A clear example is illustrated in Fig. 3 for minoxidil, a pharmaceutical used to treat alopecia. A peak corresponding to minoxidil was observed at RT 5.1 min, while a peak corresponding to its glucuronide metabolite appeared at RT 4.9 min. The glucuronide metabolite exhibited shared fragment ions with minoxidil, highlighting the structural similarities between the two forms. Similarly, other 5 chemicals such as daidzein, genistein, paracetamol, fenoprofen, and its metabolite 4-hydroxy-fenoprofen (4-OH-fenoprofen) also showed the presence of their respective glucuronide metabolites.

These contaminants, as well as the other ones found in our samples via wide-scope target screening (see “Applicability of the method” for more details), were semi-quantified using an in-house developed tool. This tool, which is currently being prepared for public release (manuscript in preparation), was based on the *ionization efficiency* strategy suggested in earlier publications [40, 41]. Notwithstanding, unlike these previous approaches, the present tool was built using matrix-matched calibration curves in urine. By incorporating specific molecular descriptors, the tool accounted for matrix effects and ionization efficiency, resulting in potentially lower errors compared to solvent-based strategies. To evaluate the performance of the semi-quantitative tool, we selected eight compounds previously detected in the samples: mono octyl phthalate, mono 2-ethylhexyl phthalate, mono butyl + isobutyl phthalate, bisphenol S, bisphenol A, nicotine, and caffeine. These chemicals were also included in the calibration curve (SI-1, Table S1), to allow for a comparison between the concentration values obtained from the semi-quantitative tool and those directly determined from the curve. The ratio between the concentrations obtained from the semi-quantitative tool and the curve ranged from 49 to 175% for all chemicals. This demonstrates that semi-quantitative results, despite not being accurate values, were in agreement with the direct determinations from the calibration curve.

The *Cap-Glu* protocol has been demonstrated to extensively deconjugate glucuronide-form compounds, as evidenced by the increase in the RT of minoxidil after deconjugation (RT: 5.1 min). Comparatively, the peak corresponding to minoxidil glucuronide completely disappeared (RT:4.9 min), indicating successful deconjugation. It is worth noting that the *Cap-Glu* samples were diluted three times compared to the *Cap* protocol samples. Minoxidil was detected in only one sample at a concentration of 700 ng·mL^−1^ using *Cap* protocol. However, when the *Cap-Glu* protocol was applied, the concentration of minoxidil was found to be 1700 ng·mL^−1^. This highlights the fact that direct analysis of minoxidil using the *Cap* protocol underestimated the total quantity of this chemical, as a portion is excreted in the glucuronide form. To account for this, it is crucial to identify and include the glucuronide forms (and other metabolites) in the calculations. This is one of the significant advantages of HRMS-based strategies. With full-scan acquisition, which compiles information on virtually all the ions present in the samples, the analysis and study of metabolites (both phase I and phase II) can be potentially conducted without the need for deconjugation steps. In the aforementioned sample, the glucuronide form of minoxidil using *Cap* protocol was determined at 422 ng·mL^−1^.

The same trend was observed for other chemicals, such as genistein, daidzein, paracetamol, or fenoprofen, for which almost the same detection frequencies were observed in both *Cap* and *Cap-Glu* (Table 1). However, one of the main limitations of using the *Cap* protocol is the quantification of glucuronides. The standards required for accurate quantification of metabolites are generally more expensive than those for parent chemicals. Also, the semi-quantitative tool used in this study had a tendency to underestimate the concentrations of glucuronides: 25–72% discrepancy (Table 1) with variations in fold change across the samples illustrated in SI-8, Figure S.4, except for paracetamol, where the semi-quantitative concentrations obtained by both *Cap* and *Cap-Glu* protocols were approximately similar (Table 1). The discrepancy in quantification can be attributed to the high predicted ionization efficiency for glucuronide metabolites, as the model did not include any of these types of chemicals. Nonetheless, further research in this direction is necessary to improve the quantification accuracy of glucuronide metabolites.

**Table 1:** Chemicals found as free and glucuronide species in the samples. Information about ionization mode, retention time (RT), *m/z*, detection frequency, and concentration range is given for both species. Finally, the ratio between average concentrations was found

			Minoxidil	Daidzein	Genistein	Paracetamol	Fenoprofen	4-OH-fenopronen^c^
Ionization mode^a^			+ ESI	+ ESI	+ ESI	+ ESI	-ESI	-ESI
RT and *m/z*	Free	RT (min)	5.1	6.5	7.3	3.5	6.9	7.4
		*m/z* (Da)	210.1349	255.0651	271.0601	152.0706	241.0870	257.0818
	Conjugated	RT (min)	4.9	4.8	5.1	3.0	4.8	5.3
		*m/z* (Da)	386.1664	431.0967	447.0916	328.1021	417.1185	433.1133
Detection frequency	Free	Cap	10%	60%	40%	20%	10%	10%
		Cap-Glu	10%	80%	80%	20%	10%	10%
	Conjugated	Cap	10%	70%	40%	20%	10%	10%
		Cap-Glu	0%	0%	0%	20%	0%	0%
Concentration range	Free	Cap	nd^b^–780	nd–102	nd–30	nd–7260	nd–3.55	nd–3.78
		Cap-Glu	nd–1770	nd–1300	nd–1870	nd–15,700	nd–630	nd–410
	Conjugated	Cap	nd–496	nd–644	nd–438	nd–18,700	nd–260	nd–100
		Cap-Glu	nd	nd	nd	nd–12,300	nd	nd
Cap/Cap-Glu			72%	57%	25%	100%	42%	25%

^a^Positive (+ ESI) and negative (-ESI) ionization mode. ^b^Non-detected (nd). ^c^4-hydroxy-fenopronen (4-OH-fenopronen)

The deconjugation step in the analysis also presents limitations. First, it significantly increases the time required for the sample processing. While a simple sample clean-up using Captiva cartridges for up to 24 samples takes around 30 min, the addition of the deconjugation step can extend the process to 4–5 h for the same number of samples (8–10 times more). When dealing with a large number of samples typically needed for reliable epidemiological results (e.g., 1000–2000 samples), this can result in excessive time consumption. Considering that similar results can be obtained using both the *Cap* and *Cap-Glu* methodologies, the longer processing time associated with the deconjugation step becomes a significant drawback. The second limitation is that samples need to be incubated at high temperatures; hence, thermally unstable chemicals could be degraded, yielding lower concentrations in the extract. In addition, the duration and temperature of the deconjugation step are critical factors in achieving complete deconjugation. For example, even after 2 h of deconjugation at 48 °C, paracetamol glucuronide was still observed in samples where it was present, and only half of the initial concentration was deconjugated. While the glucuronide peak appeared to completely disappear after 2 h for most chemicals, the potential for partial deconjugation introduces a potential source of error in the analysis of certain chemicals. Therefore, the deconjugation step has inherent limitations in terms of time consumption, thermal stability, and achieving complete deconjugation for all analytes of interest.

**Fig. 4:**
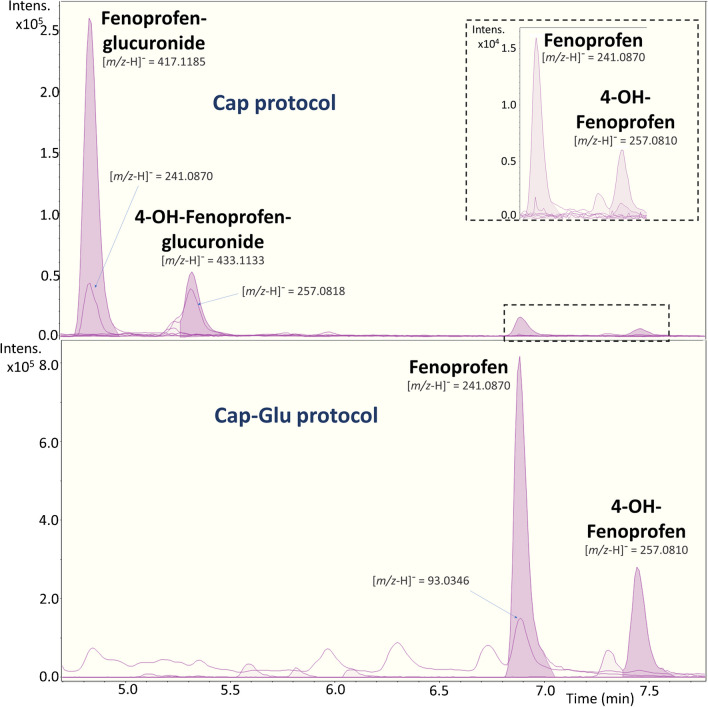
Fenoprofen (RT: 6.9 min), 4-OH-fenoprofen (RT: 7.4 min), and their glucuronide forms (RT: 4.8 and 5.3 min, respectively) chromatographic peaks for both *Cap* and *Cap-Glu* protocol. Main fragments are included

Indeed, the presence of metabolites other than glucuronides, such as hydroxylated or sulfate metabolites, can be observed in the analysis of certain compounds [46]. This was illustrated with fenoprofen or paracetamol, PhACs found in the samples by both *Cap* and *Cap-Glu* protocols (Table 1). Fenoprofen, a non-steroidal anti-inflammatory drug (NSAID), is commonly excreted in urine conjugated, mainly, as glucuronide or 4-hydroxy-fenoprofen glucuronide [47]. As expected, the glucuronide form was detected by the Cap protocol, but also hydroxy-fenoprofen (OH-fenoprofen) and hydroxy-fenoprofen glucuronide were found (Fig. 4). Parallelly, paracetamol sulfate was found in the same samples than paracetamol (Table 2). The advantage of using HRMS is that it allows for the identification of multiple chemical species in a single analytical run without the need for deconjugation steps. In this case, the wide-scope target screening approach may not have specifically targeted these hydroxy or sulfate metabolites. However, using suspect screening strategies, the detection of these metabolites becomes possible without the need for deconjugation.

**Table 2:** Chemicals found in the 10 human urine samples analyzed. Chemical concentration expressed in ng·mL^−1^

Identity	Class	Sample										Range	Median	Average	DF^d^
		1	2	3	4	5	6	7	8	9	10	ng·mL^−1^	ng·mL^−1^	ng·mL^−1^	
Cyclamic acid	Food-related chemical	6241	8006	10,150	8470	3813	4089	5806	6260	9059	9021	3813–10,150	7133	7092	100%
1-Naphthylacetic acid	plant-growth regulator	3.41	nd	14.58	nd	nd	nd	nd	1.51	1.04	2.18	nd–14.58	0.52	2.27	50%
Bisphenol S	Plastic additive	nd^c^	nd	nd	nd	nd	nd	nd	0.35	nd	nd	nd–0.35	0	0.035	10%
Bisphenol A	Plastic additive	2.81	4.2	2.86	3.02	1.3	1.49	2.75	nd	2.63	nd	nd–4.2	2.69	2.11	80%
Mono 2-ethylhexyl phthalate	Plastic additive	nd	nd	nd	0.71	nd	nd	nd	nd	nd	nd	nd–0.71	0	0.071	10%
Mono n-octyl phthalate	Plastic additive	nd	nd	nd	nd	nd	nd	nd	nd	nd	0.18	nd–0.18	0	0.018	10%
Mono isobutyl phthalate + mono n-butyl phthalate	Plastic additive	nd	0.36	0.4	nd	0.42	1.06	nd	nd	2.37	nd	nd–2.37	0.18	0.46	0.5
Methylparaben	PCPs^a^	nd	0.06	nd	0.25	0.05	0.01	0.15	0.21	nd	1.48	nd–1.48	0.055	0.22	70%
1,2-Benzisothiazolinone	Biocide	nd	7.09	41.3	9.37	nd	23.6	nd	nd	nd	4.32	nd–41.3	2.16	8.57	50%
Caffeine	Food-related chemical	nd	11.5	1445	368	31.1	110	176	nd	78	6.38	nd–1445	54.55	223	80%
Nicotinamide	Food-related chemical	161	220	96	102	110	153	91.2	91.3	132	91.7	91.2–220	106	125	100%
Nicotine	Tobacco-related chemicals	nd	nd	nd	246	nd	nd	680	90.7	79.9	nd	nd–680	0	110	40%
Nicotine-nor	Tobacco-related chemicals	nd	nd	nd	150	nd	nd	408	4.7	51.5	nd	nd–408	0	61.4	40%
Cotinine	Tobacco-related chemicals	nd	nd	nd	1427	nd	nd	2170	41	646	nd	nd–2170	0	428	40%
Anabasine	Tobacco-related chemicals	nd	nd	nd	38	nd	nd	100	0.96	16.8	nd	nd–100	0	15.6	40%
Cotinine-hydroxy	Tobacco-related chemicals	nd	nd	nd	782	nd	nd	864	10.1	375	nd	nd–864	0	203	40%
Nitenpyram	Biocide	nd	nd	nd	nd	nd	46.6	nd	nd	nd	nd	nd–46.6	0	4.66	10%
Propylparaben	PCPs	nd	19.5	24.7	8.23	9.5	40.7	nd	nd	9.4	6.01	nd–40.7	8.82	11.8	70%
Quinine	Food-related chemical	nd	nd	428	nd	nd	nd	nd	nd	145	nd	nd–428	0	57.3	20%
Quinmerac	Biocide	nd	1.4	nd	nd	nd	nd	nd	nd	1.04	1	nd–1.4	0	0.34	30%
Theobromine	Food-related chemical	nd	1124	877	929	125	877	1417	nd	30	316	nd–1417	597	570	80%
Theophylline	Food-related chemical	27.3	2977	2309	2485	3328	2345	3960	11.07	811	830	11.07–3960	2327	1908	100%
Minoxidil	PhACs^b^	nd	nd	nd	nd	nd	nd	nd	nd	nd	780	nd–780	0	78.0	10%
Minoxidil glucuronide	PhACs metabolite	nd	nd	nd	nd	nd	nd	nd	nd	nd	496	nd–496	0	49.6	10%
Daidzein	Food-related chemical	nd	102	2.78	13.8	nd	39.5	nd	0.81	nd	11.5	nd–102	1.80	17.0	60%
Daidzein glucuronide	Food-related chemical metabolite	2.6	644	10.7	85	nd	123	nd	4.78	nd	38.1	nd–644	7.74	90.8	70%
Genistein	Food-related chemical	nd	30	nd	5.91	nd	3.78	nd	nd	nd	4.04	nd–30	0	4.37	40%
Genistein glucuronide	Food-related chemical metabolite	nd	438	nd	62.7	nd	30.2	nd	nd	nd	38.3	nd–438	0	56.9	40%
Paracetamol	PhACs	836	nd	nd	nd	nd	7261	nd	nd	nd	nd	nd–7261	0	810	20%
Paracetamol glucuronide	PhAC metabolite	1158	nd	nd	nd	nd	18,770	nd	nd	nd	nd	nd–18770	0	1993	20%
Paracetamol sulfate	PhACs metabolite	4200	nd	nd	nd	nd	20,900	nd	nd	nd	nd	nd–20,900	0	2510	20%
Fenoprofen	PhACs	nd	nd	nd	nd	nd	nd	nd	nd	nd	3.55	nd–3.55	0	0.36	10%
Fenoprofen glucuronide	PhACs	nd	nd	nd	nd	nd	nd	nd	nd	nd	260	nd–260	0	26	10%
4-OH-fenoprofen	PhACs	nd	nd	nd	nd	nd	nd	nd	nd	nd	3.78	nd–3.78	0	0.38	10%
4-OH-fenoprofen glucuronide	PhACs	nd	nd	nd	nd	nd	nd	nd	nd	nd	100	nd–100	0	10	10%

^a^Personal care products (PCPs). ^b^Pharmaceutically active compounds (PhACs). ^c^Detection frequency (DF). ^d^Non-detected (nd). The following chemicals were found by suspect screening: 4-OH-fenoprofen, paracetamol sulfate as well as glucuronides

Despite the necessity of including some glucuronide compounds in the training of the semi-quantitative model to minimize errors in their semi-quantification, the advantages provided by HRMS and semi-quantification outweigh the drawbacks associated with deconjugation steps. Also, these approaches have the potential to uncover additional metabolites in urine samples, expanding the scope of analysis.

### Application of the suspect screening and semi-quantification methodology to real samples

A total of 36 exogenous chemicals (including the metabolites previously mentioned) were detected in human urine samples (Table 2). These chemicals encompass a wide range of categories such as food-related chemicals, PCPs, tobacco-related chemicals, biocides, plastic additives, plant-growth regulators, and PhACs. These chemicals were detected and semi-quantified in the samples.

Some of these chemicals have been found in almost 100% of samples. Some examples are cyclamic acid, daidzein, caffeine, theobromine, or theophylline. These chemicals are primarily ingested through food consumption, as they are naturally found in foodstuffs (such as coffee and chocolate, among others). The semi-quantitative analysis yielded concentration levels that were consistent with those previously reported in the literature. For instance, the levels of caffeine, theobromine, and theophylline were in the range of the ones reported in other studies [48, 49], and so were daidzein [50] and cyclamic acid [51].

Another possible contaminant entering through the diet was quinmerac, an herbicide which has recently attracted the attention of the scientific community, as it has been determined in urine samples under the HBM4EU project [52]. Similarly, methylparaben (MeP) and propylparaben (PrP), chemicals, which are known as endocrine-disrupting chemicals [53] and commonly used as preservatives in personal care products, have been determined in 70% of the samples, ranging between nd–1.48 ng·mL^−1^ (MeP), and between nd–40.7 ng·mL^−1^ (PrP). This high detection frequency agrees with previous reports where MeP and PrP were detected in a substantial percentage of samples, with concentrations up to 2002 ng·mL^−1^ for MeP and 256 ng·mL^−1^ for PrP [54].

Nitenpyram, a neonicotinoid insecticide commonly used to treat flea infestations on pets, was detected in only one of the samples at 46.6 ng·mL^−1^. A recent study reported a 100% detection frequency of this chemical in human urine (max. concentration: 1.1 µg·g^−1^) [55]. Similarly, 1,2-benzisothiazolinone, widely used as a preservative and antimicrobial, was found in 50% of the samples at levels up to 41.3 ng·mL^−1^. To the best of the authors’ knowledge, it has never been reported in urine samples, despite a methodology developed to specifically extract it from this matrix [56]. The extent of human exposure to these ingredients commonly found in personal care products remains unclear.

Other chemicals can enter the body through inhalation. This route of exposure was evident for tobacco-related chemicals, such as nicotine, and their main metabolites (nornicotine, cotinine, hydroxy-cotinine), as well as minor tobacco alkaloid anabasine, which were found in the same four urine samples. In addition, found levels were in the same range as in other publications, such as the recent publication from Oh et al. [57], with nicotine concentrations up to 11.375 ng·mL^−1^, cotinine up to 3353 ng·mL^−1^, 3-OH cotinine up to 18,324 ng·mL^−1^, nornicotine up to 345 ng·mL^−1^, and anabasine up to 40 ng·mL^−1^.

## Conclusions

In the present study, five extraction protocols were compared in terms of sensitivity, trueness, and matrix effects. Among them, the best-performing protocol was successfully validated for 90 chemicals in terms of sensitivity (LOQs and LODs), trueness (recoveries at 2, 10, and 50 µg L^−1^), precision (intra- and inter-day), linear and dynamic range, as well as matrix effects. The LOQs achieved were in the range from pg·mL^−1^ to ng·mL^−1^, and recoveries generally fell, between 70 and 120%. Inter- and intra-day reproducibility was also satisfactory for approximately 75% of the chemicals tested. These findings demonstrate the reliability and robustness of the methodology, supporting its suitability for the wide-scope objectives of the study.

The validated methodology was subsequently applied to a set of 10 individual urine samples to evaluate its ability to perform wide-scope target as well as suspect/non-target screening. In these samples, a total of 36 contaminants were detected, including glucuronide and hydroxylated or sulfate metabolites. The identified chemicals belonged to various categories such as food-related chemicals, personal care products, tobacco-related chemicals, biocides, plastic additives, plant-growth regulators, and pharmaceuticals. To determine their concentrations, a semi-quantitative approach was employed using a tool based on ionization efficiency. This tool was trained using urine matrix-matched calibration curves. The semi-quantitative results were compared to the accurate concentrations obtained by using standards in matrix-matched calibration curves. The concentrations determined by the semi-quantitative tool showed similarity ranging from 49 to 175% in contrast to accurate concentrations. This indicates that the semi-quantitative approach provides reasonably close estimations of the chemical concentrations when compared to the exact values obtained through standard calibration curves.

Finally, the need for a deconjugation step in the sample treatment protocol was assessed. Hence, analyte concentrations from a deconjugated extract were compared to the combined concentrations of all related species in an extract that did not undergo deconjugation. The same detection frequencies were observed by both strategies, while the Cap methodology revealed the presence of glucuronide metabolites. For some chemicals, the semi-quantitative concentrations obtained using both protocols were almost identical, highlighting the significant potential of these semi-quantitative HRMS-based strategies. To the best of the authors’ knowledge, this is the first study that compares these two protocols (with and without deconjugation), providing insights into their advantages and disadvantages, as well as demonstrating their potential application in quantification using HRMS instruments.

### Supplementary information

Below is the link to the electronic supplementary material.

Supplementary file1 (DOCX 476 KB)
