# Supplementary material for: Comprehensive profiling and semi-quantification of exogenous chemicals in human urine using HRMS-based strategies
Source: Anal Bioanal Chem. 2023 Nov 9;415(29-30):7297–313. doi: 10.1007/s00216-023-04998-9 (PMC10684428; doi:10.1007/s00216-023-04998-9)

# Supplementary Information

**Comprehensive profiling and semi-quantification of exogenous chemicals in human urine using HRMS-based strategies**

Daniel Gutiérrez-Martín^1,2,3^, Esteban Restrepo-Montes^1^, Oksana Golovko^4^, Rebeca López-Serna^2,3^, Reza Aalizadeh^5^, Nikolaos S. Thomaidis^5^, Montse Marquès^6,7^, Pablo Gago-Ferrero^1*^, Rubén Gil-Solsona^1*^

^1^Department of Environmental Chemistry, Institute of Environmental Assessment and Water Research – Severo Ochoa Excellence Center (IDAEA), Spanish Council of Scientific Research (CSIC), Barcelona 08034, Spain.

^2^Institute of Sustainable Processes (ISP), Dr. Mergelina s/n, Valladolid 47011, Spain.

^3^Department of Analytical Chemistry, Faculty of Sciences, University of Valladolid, Paseo de Belén 7, 47011 Valladolid, Spain.

^4^Department of Aquatic Sciences and Assessment, Swedish University of Agricultural Sciences (SLU), SE-75007 Uppsala, Sweden.

^5^Laboratory of Analytical Chemistry, Department of Chemistry, National and Kapodistrian University of Athens, Panepistimiopolis Zografou, 15771 Athens, Greece

^6^Universitat Rovira I Virgili, Laboratory of Toxicology and Environmental Health, School of Medicine, IISPV, Sant LLorenç 21, 43201, Reus, Catalonia, Spain.

^7^Institut d'Investigació Sanitària Pere Virgili (IISPV), Reus, Spain.

# Index

**SI-1. Reagents and materials**

**Table S1**. List of analytical standards and internal standards (IS) used in the present study.

**SI-2. UHPLC-QTOF conditions**

**Table S2**. ESI Mode (Screening Method) gradient profile for both positive and negative ionization mode.

**SI-3. Applicability of the method – Model for semi-quantification**

QSIR models for semi-quantitative analysis

QSIR workflow and models

**Fig.S1** The correlations between experimental and predicted log2IE values

**Table S3.1** Relative Log_2_IE database for model training -ESI

**Table S3.2** Relative Log_2_IE database for model training +ESI

**SI-4. Results and discussion**

**Fig.S2** Chemicals selected for method validation. Properties distribution (LogP vs RT) for both ionization modes: a) +ESI and b) -ESI

**SI-5. Method selection**

**Table S4**. Results from the comparation of all the extraction protocols.

**SI – 6. Instrumental parameters optimization**

**Fig. S3** Injection volume optimization.

**SI-7. Methods performance**

**Table S5**. Validation results for Cap protocol.

**SI-8. Deconjugation in HRMS-based strategies**

**Table S6**. Validation results for Cap-Glu protocol.

**Figure S.4.** Variations in fold change across the samples

**SI-1. Reagents and materials**

Table S1. List of analytical standards and internal standards (IS) used for the validation. IS are presented at the end of the table.

| Chemical name | Molecular formula | Uses | LogP^a^ | CAS number | IM^b^ |
| --- | --- | --- | --- | --- | --- |
| 1,2,3,4-Tetrahidro-9H-pirido[3,4-B]Indole | C11H12N2 | Pharmaceutical | 1.5 | 16502-01-5 | + |
| 1h,1h,2h,2h-perfluorooctanesulfonic acid (6:2 FTS) | C8H5F13O3S | Industrial chemical (PFAS) | 3.9 | 27619-97-2 | - |
| 2,2'-Dihydroxy-4-methoxybenzophenone | C14H12O4 | UV-Filter | 3.3 | 131-53-3 | - |
| 2-amino-Benzothiazole | C7H6N2S | Industrial chemical | 1.9 | 136-95-8 | + |
| 2-Benzothiazolesulfonic acid | C7H5NO3S2 | Industrial chemical | 1.5 | 941-57-1 | - |
| 2-Hydroxy-5-octanoylbenzoic acid | C15H20O4 | Benzoic acid derivate | 5.2 | 78418-01-6 | - |
| 3,4-Dihydroxybenzoic acid | C7H6O4 | Benzoic acid derivate | 1.1 | 99-50-3 | - |
| 3,4-Dihydroxybenzoic acid methyl ester | C8H8O4 | Benzoic acid derivate | 1.5 | 2150-43-8 | - |
| 4,4'-Dihydroxybenzophenone | C13H10O3 | UV-filter | 2.7 | 611-99-4 | - |
| 4-Aminoantipyrine | C11H13N3O | Pharmaceuticals | 0.1 | 83-07-8 | + |
| 4-Hydroxybenzoic acid-benzyl ester | C14H12O3 | Personal care product | 3.6 | 94-18-8 | - |
| 4-Hydroxybenzoic acid-isobutyl ester | C11H14O3 | Personal care product | 3.4 | 4247-02-3 | - |
| 4-Hydroxybenzoic acid-isopropyl ester | C10H12O3 | Personal care product | 2.8 | 4191-73-5 | - |
| 4-Hydroxybenzoic acid-n-butyl ester | C11H14O3 | Personal care product | 3.6 | 94-26-8 | - |
| 4-Hydroxybenzophenone | C13H10O2 | UV-filter | 3.1 | 1137-42-4 | +/- |
| Alachlor | C14H20ClNO2 | Biocide | 3.5 | 15972-60-8 | + |
| Atrazine | C8H14ClN5 | Biocide | 2.6 | 1912-24-9 | + |
| Benzophenone-1 | C13H10O3 | UV-filter | 3.2 | 131-56-6 | - |
| Benzophenone-2 | C13H10O5 | UV-filter | 2.4 | 131-55-5 | - |
| Benzophenone-3 | C14H12O3 | UV-filter | 3.6 | 131-57-7 | - |
| Benzophenone-4 | C14H12O6S | UV-filter | 2.2 | 4065-45-6 | - |
| Bisphenol A | C15H16O2 | Industrial chemical (plastic additive) | 3.3 | 80-05-7 | - |
| Bisphenol AF | C15H10F6O2 | Industrial chemical (plastic additive) | 4.5 | 1478-61-1 | - |
| Bisphenol AP | C20H18O2 | Industrial chemical (plastic additive) | 4.4 | 1571-75-1 | - |
| Bisphenol B | C16H18O2 | Industrial chemical (plastic additive) | 3.9 | 77-40-7 | - |
| Bisphenol E | C14H14O2 | Industrial chemical (plastic additive) | 3.9 | 2081-08-5 | - |
| Bisphenol F | C13H12O2 | Industrial chemical (plastic additive) | 2.9 | 620-92-8 | - |
| Bisphenol G | C21H28O2 | Industrial chemical (plastic additive) | 6.3 | 127-54-8 | - |
| Bisphenol M | C24H26O2 | Industrial chemical (plastic additive) | 6.1 | 13595-25-0 | - |
| Bisphenol P | C24H26O2 | Industrial chemical (plastic additive) | 6.1 | 2167-51-3 | - |
| Bisphenol S | C12H10O4S | Industrial chemical (plastic additive) | 1.9 | 80-09-1 | - |
| Bisphenol Z | C18H20O2 | Industrial chemical (plastic additive) | 5.4 | 843-55-0 | - |
| Bromacil | C9H13BrN2O2 | Biocide | 2.1 | 314-40-9 | + |
| Carbamazepine | C15H12N2O | Pharmaceutical | 2.5 | 298-46-4 | + |
| Carbendazim | C9H9N3O2 | Biocide | 1.5 | 10605-21-7 | + |
| Chlorpyriphos | C9H11Cl3NO3PS | Biocide | 5.3 | 2921-88-2 | + |
| Clarithromycin | C38H69NO13 | Pharmaceutical | 3.2 | 81103-11-9 | - |
| Dimethomorph | C21H22ClNO4 | Biocide | 3.9 | 110488-70-5 | + |
| Dimethyl-benzotriazole | C8H9N3 | Industrial chemical (anticorrosion agent) | 1.8 | 35899-34-4 | +/- |
| Diuron | C9H10Cl2N2O | Biocide | 2.7 | 330-54-1 | +/- |
| Di-β,β'-Chloroethylphosphoric Acid | C4H9Cl2O4P | Flame retardant | 0.2 | 3040-56-0 | - |
| Enrofloxacin | C19H22FN3O3 | pharmaceutical (veterinary use) | -0.2 | 93106-60-6 | + |
| Enzacamene | C18H22O | UV-Filter | 4.5 | 36861-47-9 | + |
| Ethyl 3,4-Dihydroxybenzoate | C9H10O4 | Food related chemical | 1.8 | 3943-89-3 | - |
| Ethyl paraben | C9H10O3 | Food additive / Personal care product | 2.5 | 120-47-8 | + |
| Flumequine | C14H12FNO3 | Biocide | 2.9 | 42835-25-6 | + |
| Gemfibrozil | C15H22O3 | Pharmaceutical | 3.8 | 25812-30-0 | - |
| Hexadecylamine | C16H35N | Industrial chemical (antistatic) | 7.4 | 143-27-1 | + |
| Lauryl diethanolamide | C16H33NO3 | Personal care product | 3.5 | 120-40-1 | + |
| Malathion | C10H19O6PS2 | Biocide | 2.4 | 121-75-5 | + |
| Mecoprop | C10H11ClO3 | Biocide | 3.1 | 93-65-2 | - |
| Metalaxyl | C15H21NO4 | Biocide | 1.6 | 57837-19-1 | + |
| Methiocarb | C11H15NO2S | Biocide | 2.9 | 2032-65-7 | + |
| Methyl-benzotriazole | C7H7N3 | Industrial chemical (anticorrosion agent) | 1.4 | 29878-31-7 | +/- |
| Molinate | C9H17NOS | Biocide | 3.2 | 2212-67-1 | + |
| Mono benzyl phthalate | C15H12O4 | Industrial chemical (phthalate) | 3.3 | 2528-16-7 | - |
| Mono cyclohexyl phthalate | C14H16O4 | Industrial chemical (phthalate) | 2.9 | 7517-36-4 | - |
| Mono isobutyl phthalate | C12H14O4 | Industrial chemical (phthalate) | 2.5 | 30833-53-5 | - |
| Mono n-butyl phthalate | C12H14O4 | Industrial chemical (phthalate) | 3.1 | 131-70-4 | - |
| Mono(2-ethyl-5-hydroxyhexyl) Phthalate | C16H22O5 | Industrial chemical (phthalate) | 2.5 | 40321-99-1 | - |
| Mono(2-ethyl-5-oxohexyl) Phthalate | C16H20O5 | Industrial chemical (phthalate) | 2.0 | 40321-98-0 | - |
| Mono-2-ethylhexyl phthalate | C16H22O4 | Industrial chemical (phthalate) | 4 | 4376-20-9 | - |
| Monooctyl Phthalate | C16H22O4 | Industrial chemical (phthalate) | 5.3 | 5393-19-1 | - |
| Monopentyl Phthalate | C13H16O4 | Industrial chemical (phthalate) | 3.7 | 24539-56-8 | - |
| N-acetyl sulfadiazine | C12H12N4O3S | Pharmaceuticals | -0.2 | 127-74-2 | + |
| N-acetyl sulfamethazine | C14H16N4O3S | Pharmaceuticals (metabolite) | 0.1 | 100-90-3 | +/- |
| N-acetyl sulfapyridine | C13H13N3O3S | Pharmaceutical (metabolite) | -0.1 | 19077-98-6 | +/- |
| Nalidixic acid | C12H12N2O3 | Pharmaceutical | 1.4 | 389-08-2 | + |
| N-desmethyl venlafaxine | C16H25NO2 | Pharmaceuticals | 3 | 149289-30-5 | + |
| Nicotine | C10H14N2 | Tobacco related chemicals | 1.2 | 54-11-5 | + |
| O-desmethyl venlafaxine | C16H25NO2 | Pharmaceutical | 2.6 | 93413-62-8 | +/- |
| Oxadiazon | C15H18Cl2N2O3 | Biocide | 4.8 | 19666-30-9 | + |
| Oxathiapiprolin | C24H22F5N5O2S | Biocide | 4.4 | 1003318-67-9 | + |
| Oxolinic acid | C13H11NO5 | Pharmaceutical | -0.2 | 14698-29-4 | + |
| Pilocarpine | C11H16N2O2 | Pharmaceutical | 1.1 | 92-13-7 | + |
| Propanil | C9H9Cl2NO | Biocide | 3.1 | 709-98-8 | + |
| Sulfadimethoxine | C12H14N4O4S | Pharmaceutical | 1.6 | 122-11-2 | +/- |
| Sulfamerazine | C11H12N4O2S | Pharmaceutical | 0.1 | 127-79-7 | + |
| Sulfamethoxazole | C10H11N3O3S | Pharmaceutical | 0.9 | 723-46-6 | + |
| Sulfamethoxypyridazine | C11H12N4O3S | Pharmaceutical | 0.3 | 80-35-3 | +/- |
| Sulfaquinoxaline | C14H12N4O2S | Pharmaceutical | 1.7 | 59-40-5 | + |
| Sulfathiazole | C9H9N3O2S2 | Pharmaceutical | 0.1 | 72-14-0 | + |
| Methyl 3,4-dichlorophenylcarbamate (SWEP) | C8H7Cl2NO2 | Biocide | 3.5 | 1918-18-9 | - |
| Terbumeton | C10H19N5O | Biocide | 3.1 | 33693-04-8 | + |
| Tonalide | C18H26O | PCPs | 5.3 | 93413-62-8 | + |
| Triclocarban | C13H9Cl3N2O | Personal care product | 5.3 | 101-20-2 | - |
| Tris(1-chloro-2-propyl) phosphate (TCPP) | C9H18Cl3O4P | Flame retardant | 2.6 | 13674-84-5 | + |
| Tris(2-chloroethyl) phosphate (TCEP) | C6H12Cl3O4P | Flame retardant | 1.3 | 51805-45-9 | + |
| Venlafaxine | C17H27NO2 | Pharmaceutical | 2.9 | 93413-69-5 | + |
| Zoxamide | C14H16Cl3NO2 | Biocide | 4.3 | 156052-68-5 | + |
| 4-Methylbenzylidene Camphor-d4 | C18H18D4O | IS | 3.8 | 1219806-41-3 | + |
| Atenolol-d7 | C14H15D7N2O3 | IS | 0.2 | 1202864-50-3 | + |
| Benzophenone-3-d5 | C14D5H7O3 | IS | NA | 1219798-54-5 | + |
| Benzotriazole-d4 | C6D4N3 | IS | 1.4 | 1185072-03-0 | +/- |
| Benzylparaben d4 | C14H8D4O3 | IS | 3.6 | 1219805-81-8 | - |
| Bis(2-ethylhexyl) phthalate-d4 | C24H34D4O4 | IS | 7.4 | 93951-87-2 | + |
| Bisphenol S-d8 | C12D8H2O4S | IS | 1.9 | 2483831-28-1 | - |
| Caffeine-d3 | C8H7D3N4O2 | IS | -0.1 | 26351-04-2 | + |
| Carbamazepine-d10 | C15H2D10N2O | IS | 2.5 | 132183-78-9 | + |
| Citalopram-d4 | C20H17D4FN2O | IS | 3.2 | 1219908-84-5 | + |
| Clothianidin-d3 | C6D₃H5ClN5O2S | IS | 1.2 | 1262776-24-8 | +/- |
| Diclofenac-d4 (phenyl-d4) | C14D4H7Cl2NO2 | IS | 4.4 | 153466-65-0 | + |
| Gemfibrozil-d6 | C15D6H16O3 | IS | 3.8 | 1184986-45-5 | - |
| Methyl paraben-d3 | C8D3H5O3 | IS | 2 | 1216543-26-8 | - |
| Metronidazole-d4 | C6D4H5N3O3 | IS | 0 | 1261392-47-5 | + |
| Nicotine-d3 | C10D3H11N2 | IS | 1.2 | 69980-24-1 | + |
| Nonylphenol-d4 | C15D4H20O | IS | 5.9 | 1173019-62-9 | - |
| Octylphenol-d17 | C14D17H5O | IS | 5.3 | 1219794-55-4 | - |
| Thiamethoxam-d3 | C8D3H7ClN5O3S | IS | 1.5 | 1294048-82-0 | + |
| Triphenylphosphate-d15 | (C6D5O)3PO | IS | 4.6 | 1173020-30-8 | + |
| Venlafaxine-d6 | C17D6H21NO2 | IS | 2.9 | 1062606-12-5 | + |

*^a^LogP computed by XLogP3 3.0, PubChem. ^b^IM : Ionization mode positive (+), negative (-) or both (+/-).*

**SI-2. UHPLC-QTOF conditions**

Table S.2. ESI Mode (Screening Method) gradient profile for both positive and negative ionization mode.

| Retention time (min) | Flow (mL·min^-1^) | %B^a^ |
| --- | --- | --- |
| 0.00 | 0.200 | 4.0 |
| 0.10 | 0.200 | 4.0 |
| 1.00 | 0.200 | 18.3 |
| 2.50 | 0.223 | 50.0 |
| 14.00 | 0.400 | 99.9 |
| 16.00 | 0.480 | 99.9 |
| 16.10 | 0.480 | 4.0 |
| 19.00 | 0.480 | 4.0 |
| 19.10 | 0.200 | 4.0 |
| 20.00 | 0.200 | 4.0 |

^a^B: organic phase.

**SI-3. Applicability of the method – Model for semi-quantification**

**QSIR models for semi-quantitative analysis**

The experimental log_2_IE values were calculated from logarithmic ratio of the slope obtained from calibration curve of individual standard divided by the slope of a reference compound according to Equation S1 (Eq.S1). The ratio of molecular weight was also considered to remove effect of measurement unit and molecular weight on log_2_IE values.[^1^](#_ENREF_1) Here, Bisphenol G was used as reference compound for –ESI; and O-desmethyl venlafaxine was used for +ESI. These compounds used as reference compounds because they showed very low slope values, a good indicator of ionization efficiency baseline, among the list of adopted emerging contaminants in **Table S3.1** and **S3.2**. Another fact that was considered during selection of a reference compounds was the ability of the selected compounds to provide an acceptable MS signal in the presence of sample matrix because there is always risk of losing the calibration curve data of the reference that is also acting as baseline IE, in case of high ME. It is worth to note that Oxolinic acid was a perfect reference compound in case of solvent based calibration curve in +ESI, however it was showing unacceptable linearity and MS signal in presence of urine matrix. Therefore, O-desmethyl venlafaxine was used as reference compound in +ESI when projecting relative log_2_IE values to urine matrix. To predict concentration of analyte of interest in real sample, the predicted/experimental ionization efficiency (log_2_IE) based on QSIR could be used as denoted in Eq. S2.

$$Exp. logIE={log}_{2}\left( \frac{{slope}_{analyte}}{{slope}_{Ref. compound}}\times\frac{{MW}_{Ref. compound}}{{MW}_{analyte}} \right) (Eq. S1)$$

$$Pred. Conc.=\frac{\left( {Peak Area}_{analyte} \right)*{MW}_{Ref. compound}}{\left[ 2^{Exp./Pred. log2IE} \right]*{slope}_{Ref. compound}*{MW}_{analyte}} (Eq. S2)$$

Although the relative log_2_IE values are dimensionless and they don’t provide the measurement unit, the unit can be derived from their calibration curve (slopes). Here, the slopes were obtained by creating the calibration curves based on concentration at µgL^-1^ unit versus peak area, thus, the predicted concentration is considered to be in µgL^-1^ unit. For the quality assurance of the semi-quantitative analysis, the framework developed in our previous study was followed.[^2^](#_ENREF_2)

**QSIR workflow and models**

The procedure of building and validating the QSIR models, based on the Ant Colony Optimization coupled to Multiple Linear Regression (ACO-MLR), can be found in our previous works [^3-5^](#_ENREF_3). Internal and external validation of the QSIR models were checked carefully using OECD principals (Regulation No. ENV/JM/MONO(2007)2) [^6^](#_ENREF_6) and protocols proposed in literature [^7^](#_ENREF_7)^,^[^8^](#_ENREF_8). These accuracy testing are discussed here briefly. Q^2^_LOO_ and Q^2^_LGO_ are cross validation techniques (leave one out and leave group out) which are a good indication of internal accuracy. Q^2^_Boot_ , often referred to as robustness measure of model, is a good measure to verify whether the model is dependent on its training set or not. In this respect, the dataset is randomly being divided several times into training and test set and then the cross-validated statistics are calculated. The high value of Q^2^_Boot_ indicates that a QSIR model is not sensitive on the adopted training set, and other combinations of compounds in the log_2_IE database can produce a relatively acceptable model. R^2^ _randomized_ and Q^2^_LOO randomized_ are the maximum squared correlation coefficient and leave-one-out cross validation values that are obtained after shuffling the molecular descriptors (X-data) for 5000 times while keeping log_2_IE values (Y-data) unshuffled. The lower values validate that the correlation between log_2_IE values with selected molecular descriptors are not by chance. Q^2^_Fn_ measures are similar to Q^2^_LOO_ concept, but they are designed exclusively for test set. The modified r^2^ value [^9^](#_ENREF_9) and the Concordance correlation coefficient (CCC) evaluating both accuracy and precision [^7^](#_ENREF_7)^,^[^10^](#_ENREF_10). CCC evaluates the degree to which pairs of observations fall on the 45° line through the origin. Generally, an appropriate model should present high values for F _Training/Test_ value, R^2^_Training/Test_, Q^2^_LOO_, Q2_Fn_, CCC _Training/Test_ and r^2^_m,_ and low RMSE values for training and test set. For the internal and external accuracy, the following acceptance threshold values were applied; for Q^2^_F1_, Q^2^_F2_, and Q^2^_F3_ greater than 0.6 (a value in accordance with Q^2^_LOO_); r^2^_m_ greater than 0.5; Q^2^_LOO_/Q^2^_LGO_/Q^2^ _BOOT_ greater than 0.6; R^2^ greater than 0.7; and cutoff value of 0.85 for CCC. William’s plot was used to find the origin of errors as well as defining the application domain of the QSIR models. More details about William plot and formulation can be found in our previous works. [^4^](#_ENREF_4)

The Eq. S3 and S4 describe the ACO-MLR models as well as descriptors (including their coefficients) to predict log_2_IE values in –ESI and +ESI, respectively.

For -ESI:

$${log}_{2}IE=+3.527\left( \pm0.6800 \right)-4.643\left( \pm0.6310 \right)GATS6i+88.55\left( \pm22.44 \right)\mathrm{VE}2_{\mathrm{Dzi}}+0.7463\left( \pm0.05266 \right)minHBint6+1.132\left( \pm0.3343 \right)ExtFP843+2.927\left( \pm0.3700 \right)ExtFP860 (Eq. S3)$$

N_train_=45, R^2^_train_=0.882, RMSE_train_=0.900, R^2^_adj_=0.868, F_train_=60.03, Q^2^_LOO_=0.844, Q^2^_LGO_=0.801, Q^2^_BOOT_=0.799, N_test_=11, R^2^_test_=0.881, RMSE_test_=0.900, rm^2^_test_ =0.823, CCC_test_=0.935, CCC_cross-validation_=0.917, CCC_train_=0.938, Q^2^_F1_=0.869, Q^2^_F2_=0.868, Q^2^_F3_=0.882, max R^2^_randomized_ = 0.231, max Q^2^_LOO randomized_ = 0.170

Where “GATS6i” is Geary autocorrelation - lag 6 / weighted by first ionization potential. This descriptor (with relative importance (RI) of 16.40%) denotes how atomic property (in this case, first ionization potential) is distributed along the topological structure (or maximum distance of “D” (here it is D=6) in the graph). [^11^](#_ENREF_11) VE2_Dzi (with RI of 9.46%) is average coefficient sum of the last eigenvector from Barysz matrix / weighted by first ionization potential. minHBint6 (with RI of 54.52%) is minimum E-State descriptors of strength for potential hydrogen bonds of path length 6. ExtFP843 (with RI of 1.74%) and ExtFP860 (with RI of 17.88%) are Chemistry Development Kit (CDK) extended fingerprint which extends the Fingerprinter with additional bits describing ring features.

For +ESI:

$${log}_{2}IE=+6.698\left( \pm0.837 \right)-4.063\left( \pm0.904 \right)GATS5i-2.128\left( \pm0.437 \right)\mathrm{SHsOH}+5.825\left( \pm0.850 \right)\mathrm{minHBd}-17.242\left( \pm5.413 \right)ETA dEpsilon B-3.554\left( \pm0.472 \right)GraphFP451+2.108\left( \pm0.405 \right)GraphFP587-2.616\left( \pm0.431 \right)PubchemFP392 (Eq. S4)$$

N_train_=62, R^2^_train_=0.775, RMSE_train_=1.26, R^2^_adj_=0.746, F_train_=26.60, Q^2^_LOO_=0.700, Q^2^_LGO_=0.698, Q^2^_BOOT_=0.688, N_test_=17, R^2^_test_=0.780, RMSE_test_=1.13, rm^2^_test_ =0.642, CCC_test_=0.867, CCC_cross-validation_=0.830, CCC_train_=0.873, Q^2^_F1_=0.757, Q^2^_F2_=0.757, Q^2^_F3_=0.826, max R^2^_randomized_ = 0.229, max Q^2^_LOO randomized_ = 0.139

Where “GATS5i” is Geary autocorrelation - lag 5 / weighted by first ionization potential with RI of 10.45%. SHsOH is sum of atom-type H E-State in “-OH” with RI of 7.73%. minHBd is the minimum E-States for (strong) hydrogen bond donors with RI of 22.13%. ETA_dEpsilon_B is a measure of contribution of unsaturation and shows RI of 7.77%. GraphFP451 (with RI of 32.98%) and GraphFP587 (with RI of 8.39%) are CDK chemical graph fingerprint and they do not take bond orders into account. PubchemFP392 (with RI of 10.55%) is PubChem fingerprint that investigates the presence of atom nearest neighbor predefined patterns (PubchemFP392 is defined as “N(~C)(~C)(~H)”), regardless of bond order (denoted by "~") or count.

**Figure S1** show the degree of correlation between predicted log_2_IE values from Eq. S3- S4 and experimental log_2_IE values as well as outlier analysis. All chemicals are within the application domain of models (**Figure S1 C&D**) and there are not any substantial cases resulting increase of residuals due to either chemical structural diversity (shown as hat values) or indifferent ionization behavior (exploited as error observed in terms of standardized residuals). The further validation as well as the utility of the models are discussed in urine samples.


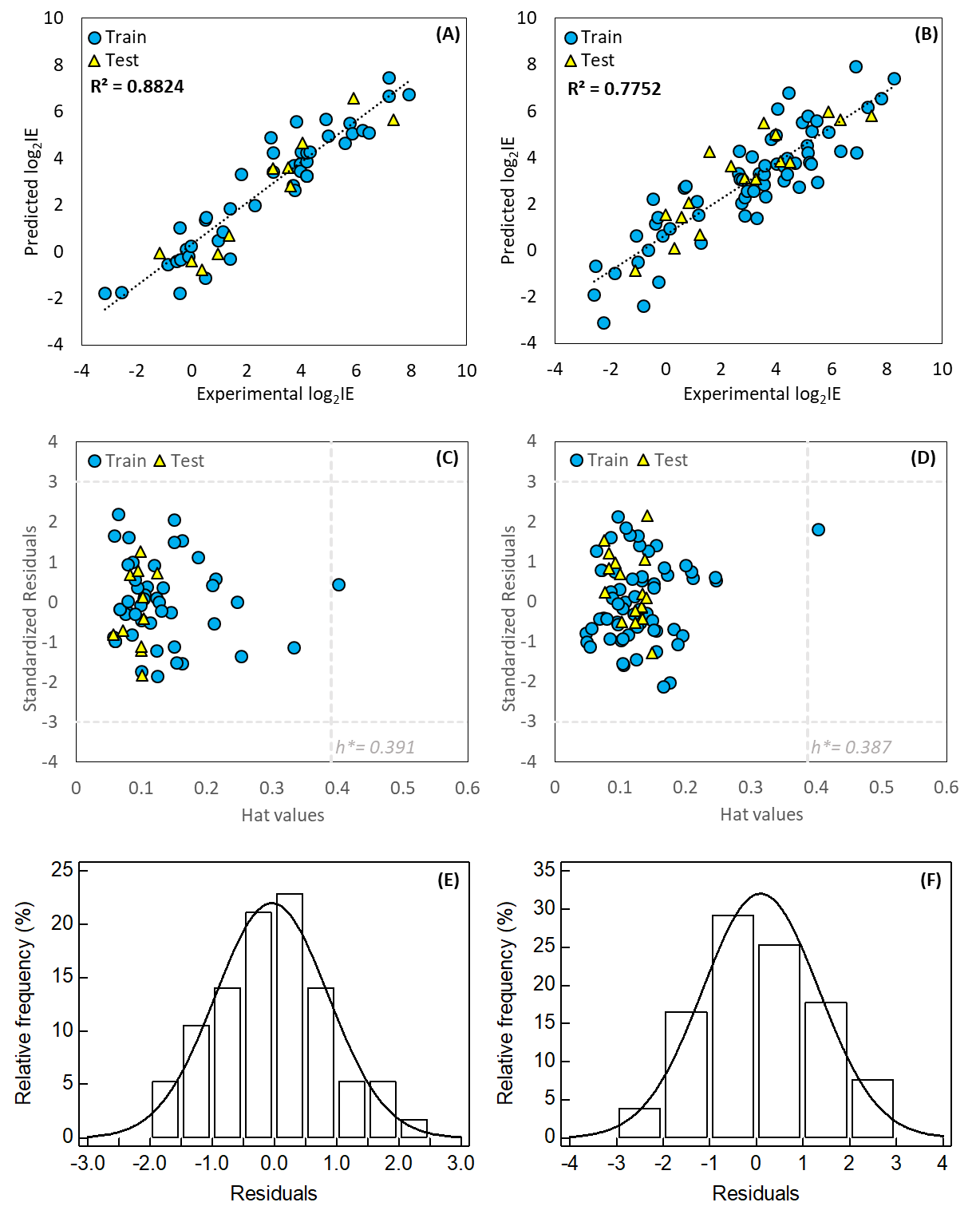


**Fig. S1** The correlations between experimental and predicted log2IE values are depicted for (A) –ESI and (B) +ESI. These two plots show the agreement between predicted and experimental ionization efficiency values for training and test set which are acceptable according to model development guidelines. [^12^](#_ENREF_12) (C) and (D) are Williams plot which provide application domain of the developed QSIR models for –ESI and +ESI, respectively. This plot shows if increase in standardized residual is due to structural diversity or purely from experimental ionization efficiency. In other words, it provides information about origin of error for individual compound in the model. [^12^](#_ENREF_12) The distributions of error between predicted and experimental log_2_IE values are depicted for (E)-ESI and (F) +ESI, respectively. More than 50% data show error below absolute 1.0 (log_2_IE) unit.

**References**

^1^ Cherkasov, A.; Muratov, E. N.; Fourches, D.; Varnek, A.; Baskin, I. I.; Cronin, M.; Dearden, J.;Gramatica, P.; Martin, Y. C.; Todeschini, R.; Consonni, V.; Kuz’min,V. E.; Cramer, R.; Benigni, R.; Yang, C.;Rathman, J.; Terfloth, L.; Gasteiger, J.; Richard, A.; Tropsha, A.Journal of Medicinal Chemistry2014,57,4977-5010.

^2^ Aalizadeh, R.; Nikolopoulou, V.; Alygizakis, N.; Slobodnik, J.; Thomaidis, N. S.AnalyticalandBioanalytical Chemistry2022,414, 7435-7450.

^3^ Aalizadeh, R.; Panara, A.; Thomaidis, N. S.Journal of the American Society for Mass Spectrometry2021,32, 1412-1423.

^4^ Aalizadeh, R.; Thomaidis, N. S.; Bletsou, A. A.; Gago-Ferrero, P.Journal of chemical information andmodeling2016,56, 1384-1398.

^5^ Aalizadeh, R.; Alygizakis, N. A.; Schymanski, E. L.; Krauss, M.; Schulze, T.; Ibáñez, M.; McEachran, A.D.; Chao, A.; Williams, A. J.; Gago-Ferrero, P.; Covaci, A.; Moschet, C.; Young, T. M.; Hollender, J.;Slobodnik, J.; Thomaidis, N. S.Analytical Chemistry2021,93, 11601-11611.

^6^ Development, O. f. E. C. o. a., 2007.

^7^ Chirico, N.; Gramatica, P.Journal of chemical information and modeling2011,51, 2320-2335.

^8^ Chirico, N.; Gramatica, P.Journal of chemical information and modeling2012,52, 2044-2058.

^9^ Roy, P. P.; Roy, K.QSAR & Combinatorial Science2008,27, 302-313.

^10^ Lin, L.Biometrics1989,45, 255-268.

^11^ Todeschini, R.; Consonni, V.Handbook of Molecular Descriptors; WILEY‐VCH Verlag GmbH:Germany, 2000, p 1-667.

^12^ Gramatica, P.QSAR & Combinatorial Science2007,26, 694-701.

**Table S3.1.** Relative Log_2_IE database for exposomics study of urine samples, including experimental and predicted values, developed for –ESI

| **ID** | **Name** | **Group** | **CAS RN** | **Experimental log2IE** | **Predicted log2IE** |
| --- | --- | --- | --- | --- | --- |
| 1 | PFBS | Training set | 375-73-5 | 7.179 | 6.722 |
| 2 | 2-Hydroxy-5-octanoylbenzoic acid | Training set | 78418-01-6 | 7.171 | 7.507 |
| 3 | 4,4'-Dihydroxybenzophenone | Training set | 611-99-4 | 6.453 | 5.119 |
| 4 | Mono(2-ethyl-5-oxohexyl) Phthalate | Test set | 40321-98-0 | 3.607 | 2.851 |
| 5 | BPS | Training set | 80-09-1 | 2.956 | 4.288 |
| 6 | Ethyl 3,4-Dihydroxybenzoate | Training set | 3943-89-3 | 5.846 | 5.096 |
| 7 | 4-Hydroxybenzoic acid-benzyl ester (Benz. paraben) | Training set | 94-18-8 | 5.752 | 5.558 |
| 8 | Propylparaben | Test set | 94-13-3 | 7.362 | 5.684 |
| 9 | Methyl paraben | Training set | 99-76-3 | 4.976 | 4.997 |
| 10 | Benzophenone-2 | Test set | 131-55-5 | 3.506 | 3.626 |
| 11 | 3,4-Dihydroxybenzoic Acid Methyl Ester | Training set | 2150-43-8 | 4.867 | 5.732 |
| 12 | Monopentyl Phthalate | Test set | 24539-56-8 | 4.027 | 4.674 |
| 13 | Diuron | Training set | 330-54-1 | 1.404 | 1.896 |
| 14 | Mono-2-ethylhexyl phthalate | Training set | 4376-20-9 | 4.185 | 3.279 |
| 15 | Monooctyl Phthalate | Training set | 5393-19-1 | 4.185 | 4.279 |
| 16 | Phthalic acid, mono-2-ethylhexyl ester | Training set | 4376-20-9 | 4.185 | 3.279 |
| 17 | Mefenamic acid | Training set | 61-68-7 | 3.756 | 2.656 |
| 18 | Benzophenone-1 | Training set | 131-56-6 | 3.791 | 5.620 |
| 19 | Triclocarban | Training set | 101-20-2 | 3.965 | 4.326 |
| 20 | Methyl-benzotriazole | Training set | 136-85-6 | 5.586 | 4.686 |
| 21 | 4-nitrophenol | Training set | 100-02-7 | 7.901 | 6.782 |
| 22 | Mono isobutyl phthalate | Training set | 30833-53-5 | 3.962 | 3.804 |
| 23 | Mono n-butyl phthalate | Training set | 131-70-4 | 3.962 | 3.733 |
| 24 | Phthalic acid, monoisobutyl ester | Training set | 30833-53-5 | 3.962 | 3.804 |
| 25 | Phthalic acid, mono-n-butyl ester | Training set | 131-70-4 | 3.962 | 3.494 |
| 26 | Dinoterb | Test set | 1420-07-1 | 5.902 | 6.619 |
| 27 | Mono cyclohexyl phthalate | Training set | 7517-36-4 | 3.692 | 2.879 |
| 28 | SWEP | Training set | 1918-18-9 | 2.288 | 2.009 |
| 29 | Mono benzyl phthalate | Test set | 2528-16-7 | 2.951 | 3.588 |
| 30 | Phthalic acid, mono-benzyl ester | Training set | 2528-16-7 | 2.951 | 3.465 |
| 31 | Umbelliferone | Training set | 93-35-6 | 4.299 | 4.308 |
| 32 | Mecoprop | Training set | 93-65-2 | -3.162 | -1.791 |
| 33 | N-acetyl sulfamethazine | Test set | 100-90-3 | 1.372 | 0.710 |
| 34 | Salicylamide | Training set | 65-45-2 | 3.728 | 3.738 |
| 35 | N-acetyl sulfapyridine | Training set | 19077-98-6 | 1.117 | 0.880 |
| 36 | 4-Hydroxybenzophenone | Training set | 1137-42-4 | 6.216 | 5.228 |
| 37 | Sulfamethoxazole | Test set | 723-46-6 | 0.368 | -0.737 |
| 38 | Mono(2-ethyl-5-hydroxyhexyl) Phthalate | Training set | 40321-99-1 | 2.871 | 4.932 |
| 39 | Sulfadimethoxine | Training set | 122-11-2 | 0.485 | 1.405 |
| 40 | Sulfathiazole | Training set | 72-14-0 | -0.432 | 1.079 |
| 41 | Bisphenol M | Test set | 13595-25-0 | 0.944 | -0.074 |
| 42 | Bisphenol P | Training set | 2167-51-3 | 0.944 | 0.528 |
| 43 | 2,2?-Methylenebis(6-tert-butyl-4-methyl-phenol) | Training set | 119-47-1 | 1.404 | -0.269 |
| 44 | Bisphenol AP | Training set | 1571-75-1 | -0.565 | -0.387 |
| 45 | 2,2'-Dihydroxy-4-methoxybenzophenone | Training set | 131-53-3 | 4.183 | 3.921 |
| 46 | 2-hydroxybenzothiazole | Training set | 934-34-9 | 0.511 | 1.495 |
| 47 | Ketoprofen | Training set | 22071-15-4 | -0.114 | -0.177 |
| 48 | Mono(5-carboxy-2-ethylpentyl) Phthalate | Training set | 40809-41-4 | 1.795 | 3.346 |
| 49 | BPF | Training set | 620-92-8 | -0.396 | -0.312 |
| 50 | MCPA | Training set | 94-74-6 | -0.041 | 0.291 |
| 51 | Benzophenone-3 | Training set | 131-57-7 | -0.200 | 0.129 |
| 52 | Bisphenol G | Test set | 127-54-8 | 0.000 | -0.382 |
| 53 | 2-Benzothiazolesulfonic acid | Training set | 941-57-1 | 0.496 | -1.098 |
| 54 | Bisphenol Z | Training set | 843-55-0 | -0.868 | -0.522 |
| 55 | Bisphenol E | Test set | 2081-08-5 | -1.177 | -0.018 |
| 56 | O-desmethyl venlafaxine | Training set | 93413-62-8 | -2.569 | -1.739 |

**Table S3.2.** Relative Log_2_IE database for exposomics study of urine samples, including experimental and predicted values, developed for +ESI

| **ID** | **Name** | **Group** | **CASRN** | **Experimental log2IE** | **Predicted log2IE** |
| --- | --- | --- | --- | --- | --- |
| 1 | Sulfadiazine | Training set | 68-35-9 | 4.026 | 6.158 |
| 2 | Carbamazepine | Test set | 298-46-4 | 7.433 | 5.840 |
| 3 | Tris(2-chloroisopropyl) phosphate | Training set | 13674-84-5 | 5.128 | 5.837 |
| 4 | Benzotriazole | Training set | 95-14-7 | 6.875 | 7.966 |
| 5 | Monobenzyl phthalate (MP) (1,2-Benzenedicarbozylic acid, mono(phenylmethyl)ester) | Training set | 2528-16-7 | 2.658 | 4.313 |
| 6 | Dimethyl-benzotriazole | Training set | 4184-79-6 | 8.247 | 7.462 |
| 7 | 4,4'-Dihydroxybenzophenone | Training set | 611-99-4 | 3.607 | 2.367 |
| 8 | N,N-Dimethylaniline | Training set | 121-69-7 | 2.843 | 2.317 |
| 9 | 4-Aminoantipyrine | Training set | 83-07-8 | 6.895 | 4.264 |
| 10 | Enzacamene | Training set | 36861-47-9 | 4.387 | 4.009 |
| 11 | Tebuconazole | Training set | 107534-96-3 | 5.252 | 3.775 |
| 12 | Tetradecylamine | Training set | 2016-42-4 | 5.266 | 5.197 |
| 13 | Flumequine | Test set | 42835-25-6 | 6.325 | 5.676 |
| 14 | 4-formylaminoantipyrine | Training set | 1672-58-8 | -0.390 | 1.195 |
| 15 | Oxolinic acid | Training set | 14698-29-4 | 2.765 | 3.079 |
| 16 | Diazinon | Training set | 333-41-5 | 4.253 | 3.669 |
| 17 | Triethyl phosphate | Training set | 78-40-0 | 6.313 | 4.332 |
| 18 | 1,2,3,4-Tetrahidro-9H-pirido[3,-B]Indole | Training set | 16502-01-5 | 4.810 | 2.770 |
| 19 | Methyl-benzotriazole (No fragmentation available) | Training set | 136-85-6 | 7.797 | 6.606 |
| 20 | Carbamazepine-10,11-epoxy | Test set | 36507-30-9 | 5.884 | 5.996 |
| 21 | Nalidixic acid | Training set | 389-08-2 | 7.286 | 6.228 |
| 22 | Mefenamic acid | Training set | 61-68-7 | 5.483 | 2.984 |
| 23 | Sulfadimethoxine | Training set | 122-11-2 | 5.450 | 5.627 |
| 24 | Diclofenac | Training set | 15307-86-5 | 2.840 | 1.537 |
| 25 | Clarithromycin | Training set | 81103-11-9 | 3.272 | 1.431 |
| 26 | 4-Hydroxybenzophenone | Training set | 1137-42-4 | 4.659 | 3.794 |
| 27 | O-desmethyl venlafaxine | Test set | 93413-62-8 | 0.000 | 1.576 |
| 28 | Nicotine | Test set | 22083-74-5 | 4.164 | 3.875 |
| 29 | 2-ethylhexyl 4-(dimethyl-amino)benzoate | Training set | 21245-02-3 | 3.171 | 2.618 |
| 30 | Ketoprofen | Training set | 22071-15-4 | 5.186 | 3.855 |
| 31 | N-acetyl sulfamethazine | Training set | 100-90-3 | 4.000 | 3.776 |
| 32 | Hexadecylamine | Training set | 143-27-1 | 5.888 | 5.154 |
| 33 | Benzophenone-3 | Training set | 131-57-7 | 4.378 | 3.340 |
| 34 | Lauryl diethanolamide | Training set | 120-40-1 | 4.935 | 5.559 |
| 35 | 2-Hydroxy-5-octanoylbenzoic acid | Training set | 78418-01-6 | 0.709 | 2.803 |
| 36 | Drometrizole | Test set | 2440-22-4 | 1.230 | 0.700 |
| 37 | Propanil | Training set | 709-98-8 | 1.169 | 1.564 |
| 38 | Sulfamerazine | Training set | 127-79-7 | 4.440 | 6.813 |
| 39 | Sulfamethoxazole | Training set | 723-46-6 | 3.814 | 4.837 |
| 40 | Diuron | Test set | 330-54-1 | 0.831 | 2.088 |
| 41 | Sulfamethoxypyridazine | Training set | 80-35-3 | 5.104 | 4.550 |
| 42 | Malathion | Test set | 121-75-5 | 1.587 | 4.273 |
| 43 | Sulfathiazole | Training set | 72-14-0 | 2.951 | 2.588 |
| 44 | Tris(2-chloroethyl)phosphate | Training set | 115-96-8 | 3.103 | 4.075 |
| 45 | Benzophenone-1 | Training set | 131-56-6 | 2.728 | 2.086 |
| 46 | Tri-o-tolyl phosphate | Training set | 78-30-8 | 1.280 | 0.365 |
| 47 | Monopentyl Phthalate | Training set | 24539-56-8 | 3.985 | 5.025 |
| 48 | Enrofloxacin | Training set | 93106-60-6 | 3.582 | 3.697 |
| 49 | Atrazine | Test set | 1912-24-9 | 3.265 | 3.110 |
| 50 | Tributyl phosphate (TNBP) | Test set | 126-73-8 | 2.858 | 3.148 |
| 51 | Phthalic acid, mono-cyclohexyl ester | Test set | 7517-36-4 | 3.535 | 5.513 |
| 52 | Oxathiapiprolin | Training set | 1003318-67-9 | -0.110 | 0.674 |
| 53 | N-desmethyl venlafaxine | Training set | 149289-30-5 | -0.631 | 0.048 |
| 54 | Sebuthylazine | Training set | 7286-69-3 | 3.537 | 2.880 |
| 55 | Terbutylazine | Training set | 5915-41-3 | 3.537 | 3.308 |
| 56 | Dimethomorph | Test set | 110488-70-5 | 0.577 | 1.460 |
| 57 | Triclocarban | Training set | 101-20-2 | -1.081 | 0.681 |
| 58 | Umbelliferone | Training set | 93-35-6 | 4.258 | 3.061 |
| 59 | 2-(5-tert-butyl-2-hydroxyphenyl)-benzotriazole | Test set | 3147-76-0 | 0.310 | 0.128 |
| 60 | DEET | Test set | 134-62-3 | 4.498 | 3.831 |
| 61 | N-acetyl sulfadiazine | Training set | 127-74-2 | 2.621 | 3.347 |
| 62 | Molinate | Training set | 2212-67-1 | 1.110 | 2.155 |
| 63 | N-acetyl sulfapyridine | Training set | 19077-98-6 | 3.371 | 3.372 |
| 64 | Zoxamide | Training set | 156052-68-5 | -0.475 | 2.256 |
| 65 | Atrazine-desethyl | Training set | 6190-65-4 | 5.120 | 4.250 |
| 66 | Cetrimonium | Training set | 505-86-2 | 0.673 | 2.745 |
| 67 | Benzophenone-2 | Training set | 131-55-5 | -2.531 | -0.642 |
| 68 | 2-(2H-benzotriazol-2yl)-4-(1,1,3,3-tetramethylbutyl)phenol | Training set | 3147-75-9 | -1.854 | -0.946 |
| 69 | 4-acetamidoantopyrine | Training set | 519-98-2 | 0.155 | 0.976 |
| 70 | Benzothiazole | Training set | 95-16-9 | -0.823 | -2.375 |
| 71 | 2-(2H-benzotriazol-2yl)-4-methyl-6-(2-propenyl)phenol | Test set | 2170-39-0 | -1.092 | -0.846 |
| 72 | 2-Hydroxybenzothiazole (OHBT) | Training set | 934-34-9 | -2.596 | -1.890 |
| 73 | Bisphenol S (4,4?-Sulfonyldiphenol) | Training set | 80-09-1 | -0.273 | -1.305 |
| 74 | Metalaxyl | Training set | 57837-19-1 | 2.683 | 3.114 |
| 75 | Monooctyl Phthalate | Test set | 5393-19-1 | 3.965 | 5.048 |
| 76 | Mono(5-carboxy-2-ethylpentyl) Phthalate | Test set | 40809-41-4 | 2.355 | 3.678 |
| 77 | Acibenzolar-S-methyl | Training set | 135158-54-2 | -2.260 | -3.103 |
| 78 | Benzophenone | Training set | 119-61-9 | -0.294 | 1.481 |
| 79 | Dodecamethylpentasiloxane | Training set | 141-63-9 | -1.015 | -0.444 |

**SI-4. Results and discussion**


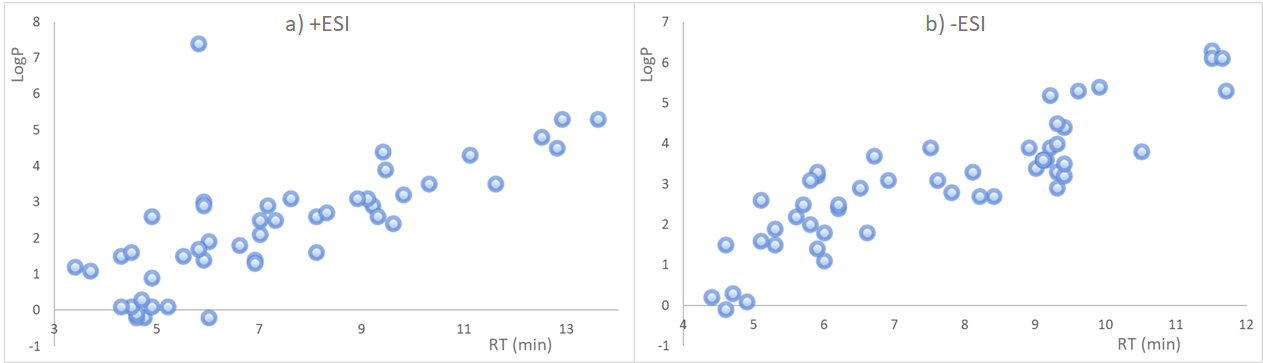
**Fig.S2** Chemicals selected for method validation. Properties distribution (LogP vs RT) for both ionization modes: a) +ESI and b) -ESI

**SI-5. Method selection**

**Table S4**. Results from the comparation of all the extraction protocols.

|  | Extraction efficiency | | | | | ME%^a^ | | | | | LOD ^b^ | | | | | IM^c^ |
| --- | --- | --- | --- | --- | --- | --- | --- | --- | --- | --- | --- | --- | --- | --- | --- | --- |
| Chemicals name | **Cent** | **Glu** | **SPE** | **Cap** | **Cap-Glu** | **Cent** | **Glu** | **SPE** | **Cap** | **Cap-Glu** | **Cent** | **Glu** | **SPE** | **Cap** | **Cap-Glu** |  |
| 1,2,3,4-Tetrahidro-9H-pirido[3,4-B]Indole | 180 | 96 | NA^d^ | 93 | 101 | 2 | 13 | 0 | 11 | 17 | 2.37 | 0.63 | NA | 0.60 | 0.55 | + |
| 1h,1h,2h,2h-perfluorooctanesulfonic acid (6:2 FTS) | 115 | 135 | NA | 137 | 59 | 119 | 94 | 0 | 116 | 147 | 0.01 | 0.03 | NA | 0.02 | 0.01 | - |
| 2,2'-Dihydroxy-4-methoxybenzophenone | 100 | 55 | 52 | 64 | 33 | 124 | 77 | 2 | 83 | 104 | 0.38 | 0.55 | 4.50 | 0.55 | 0.29 | - |
| 2-amino-benzothiazole | 116 | 123 | 80 | 107 | 115 | 22 | 51 | 1 | 24 | 49 | 0.08 | 0.03 | 0.29 | 0.06 | 0.04 | + |
| 2-Benzothiazolesulfonic acid | 109 | 132 | 1391 | 94 | 102 | 9 | 14 | 0 | 8 | 18 | 0.25 | 0.20 | 0.72 | 0.27 | 0.19 | - |
| 2-Hydroxy-5-octanoylbenzoic acid | 112 | 136 | 40 | 77 | 81 | 137 | 117 | 1 | 133 | 149 | 0.01 | 0.01 | 0.87 | 0.01 | 0.01 | - |
| 3,4-Dihydroxybenzoic acid | 115 | 135 | NA | 76 | 105 | 51 | 70 | 0 | 56 | 86 | 2.73 | 2.73 | NA | 2.73 | 2.14 | - |
| 3,4-Dihydroxybenzoic Acid Methyl Ester | 106 | 134 | 7 | 79 | 123 | 51 | 71 | 1 | 61 | 84 | 0.21 | 0.08 | 2.25 | 0.21 | 0.09 | - |
| 4,4'-Dihydroxybenzophenone | 102 | 133 | 61 | 77 | 76 | 75 | 68 | 1 | 62 | 104 | 0.02 | 0.02 | 0.53 | 0.02 | 0.02 | - |
| 4-Aminoantipyrine | 122 | 134 | 0 | 100 | 88 | 28 | 52 | 1 | 25 | 52 | 0.09 | 0.06 | 1.50 | 0.04 | 0.05 | + |
| 4-Hydroxybenzoic acid-benzyl ester | 108 | 130 | 56 | 62 | 65 | 101 | 79 | 1 | 90 | 127 | 0.03 | 0.04 | 1.10 | 0.03 | 0.03 | - |
| 4-Hydroxybenzoic acid-isobutyl ester | 107 | 138 | 41 | 106 | 117 | 97 | 78 | 2 | 64 | 92 | 0.03 | 0.05 | 0.62 | 0.05 | 0.04 | - |
| 4-Hydroxybenzoic acid-isopropyl ester | 108 | 139 | 63 | 150 | 203 | 117 | 105 | 5 | 60 | 83 | 0.03 | 0.04 | 0.73 | 0.07 | 0.05 | - |
| 4-Hydroxybenzoic acid-n-butyl ester | 107 | 130 | 29 | 92 | 108 | 99 | 76 | 2 | 73 | 95 | 0.01 | 0.02 | 0.32 | 0.02 | 0.02 | - |
| 4-Hydroxybenzophenone | 97 | 133 | 18 | 77 | 89 | 61 | 62 | 3 | 57 | 92 | 0.56 | 1.07 | 8.18 | 1.00 | 0.08 | +/**-** |
| Alachlor | 113 | 69 | NA | 128 | 81 | 102 | 53 | 0 | 68 | 48 | 1.80 | 0.86 | NA | 1.30 | 1.30 | + |
| Atrazine | 109 | 130 | NA | 109 | 112 | 38 | 55 | 0 | 33 | 51 | 0.96 | 0.50 | NA | 0.79 | 0.59 | + |
| Benzophenone-1 | 104 | 136 | 74 | 81 | 103 | 19 | 34 | 2 | 19 | 38 | 0.19 | 0.13 | 1.70 | 0.19 | 0.11 | - |
| Benzophenone-2 | 104 | 131 | NA | 85 | 95 | 21 | 34 | 0 | 21 | 42 | 0.47 | 0.17 | NA | 0.43 | 0.16 | - |
| Benzophenone-3 | 108 | 130 | 56 | 62 | 65 | 101 | 79 | 1 | 90 | 127 | 0.03 | 0.04 | 1.10 | 0.03 | 0.03 | - |
| Benzophenone-4 | 86 | 121 | NA | 86 | 113 | 91 | 85 | 0 | 77 | 98 | 0.70 | 0.63 | NA | 0.58 | 0.56 | - |
| Bisphenol A | 117 | 126 | 36 | 73 | 86 | 50 | 58 | 3 | 51 | 82 | 0.79 | 0.57 | 4.74 | 1.00 | 0.31 | - |
| Bisphenol AF | 114 | 131 | 40 | 11 | 26 | 68 | 82 | 2 | 79 | 124 | 0.11 | 0.10 | 2.05 | 0.10 | 0.06 | - |
| Bisphenol AP | 108 | 131 | 100 | 19 | 36 | 79 | 67 | 0 | 66 | 100 | 0.16 | 0.18 | NA | 0.25 | 0.14 | - |
| Bisphenol B | 107 | 125 | 48 | 70 | 67 | 70 | 63 | 3 | 56 | 95 | 0.33 | 0.37 | 5.29 | 0.50 | 0.20 | - |
| Bisphenol E | 106 | 137 | NA | 81 | 111 | 70 | 62 | 0 | 56 | 76 | 0.31 | 0.58 | NA | 0.43 | 0.45 | - |
| Bisphenol F | 111 | 60 | NA | 68 | 40 | 100 | 75 | 0 | 74 | 97 | 1.32 | 2.37 | NA | 1.88 | 1.50 | - |
| Bisphenol G | 80 | 80 | 21 | 2 | 3 | 49 | 69 | 4 | 48 | 118 | 0.12 | 0.10 | 1.25 | 0.18 | 0.05 | - |
| Bisphenol M | 77 | 96 | 22 | 9 | 3 | 71 | 69 | 3 | 45 | 152 | 0.05 | 0.05 | 1.53 | 0.08 | 0.03 | - |
| Bisphenol P | 78 | 95 | 23 | 9 | 3 | 70 | 69 | 3 | 45 | 151 | 0.04 | 0.05 | 1.53 | 0.08 | 0.03 | - |
| Bisphenol S | 107 | 134 | NA | 75 | 105 | 15 | 29 | 0 | 15 | 32 | 0.26 | 0.11 | NA | 0.22 | 0.11 | - |
| Bisphenol Z | 112 | 134 | 42 | 38 | 39 | 97 | 75 | 2 | 82 | 126 | 0.18 | 0.26 | 6.92 | 0.28 | 0.19 | - |
| Bromacil | 129 | 139 | NA | 76 | 94 | 22 | 48 | 0 | 33 | 56 | 2.90 | 2.90 | NA | 2.73 | 2.50 | + |
| Carbamazepine | 109 | 139 | 225 | 94 | 96 | 48 | 74 | 1 | 52 | 81 | 0.02 | 0.01 | 0.73 | 0.02 | 0.01 | + |
| Carbendazim | 115 | 129 | 96 | 69 | 100 | 26 | 60 | 2 | 44 | 72 | 0.08 | 0.03 | 0.59 | 0.03 | 0.02 | + |
| Chlorpyriphos | 65 | 0 | -2 | 10 | 0 | 90 | 97 | 15 | 92 | 180 | 0.64 | 0.60 | 1.02 | 0.65 | 0.32 | + |
| Clarithromycin | 102 | 143 | NA | 174 | 58 | 27 | 41 | 0 | 12 | 47 | 5.00 | 3.91 | NA | 10.00 | 4.29 | - |
| Dimethomorph | 108 | 138 | 371 | 74 | 89 | 76 | 83 | 7 | 78 | 90 | 1.43 | 1.41 | 4.09 | 1.36 | 1.30 | + |
| Dimethyl-benzotriazole | 101 | 128 | 63 | 80 | 116 | 57 | 48 | 5 | 52 | 64 | 0.05 | 0.06 | 0.50 | 0.05 | 0.04 | +/**-** |
| Diuron | 113 | 145 | NA | 89 | 99 | 44 | 59 | 0 | 48 | 69 | 0.29 | 0.22 | NA | 0.26 | 0.21 | **+**/- |
| Di-β,β'-Chloroethylphosphoric Acid | 110 | 135 | NA | 105 | 116 | 52 | 127 | 0 | 46 | 150 | 0.52 | 0.16 | NA | 0.55 | 0.12 | - |
| Enrofloxacin | 111 | 102 | 401 | 69 | 35 | 94 | 276 | -1 | 113 | 584 | 0.20 | 0.12 | 2.22 | 0.16 | 0.06 | + |
| Enzacamene | 70 | 0 | 66 | 1 | 1 | 52 | 66 | 10 | 43 | 93 | 0.10 | 0.06 | 0.38 | 0.09 | 0.06 | + |
| Ethyl 3,4-Dihydroxybenzoate | 112 | 135 | 250 | 82 | 108 | 61 | 77 | -3 | 62 | 101 | 0.11 | 0.08 | 0.39 | 0.11 | 0.07 | - |
| Ethyl paraben | 96 | 108 | 4 | 197 | 175 | 86 | 101 | -42 | 76 | 64 | 10.00 | 7.50 | 15.00 | 15.00 | 10.00 | + |
| Flumequine | 109 | 144 | -14 | 88 | 67 | 47 | 62 | 1 | 49 | 99 | 0.03 | 0.02 | 0.95 | 0.02 | 0.01 | + |
| Gemfibrozil | 107 | 127 | 32 | 61 | 87 | 65 | 69 | 2 | 85 | 103 | 0.47 | 0.59 | 6.92 | 0.75 | 0.42 | - |
| Hexadecylamine | 118 | 137 | 50 | 34 | 78 | 46 | 70 | 4 | 50 | 79 | 0.01 | 0.01 | 0.18 | 0.01 | 0.01 | + |
| Lauryl diethanolamide | 76 | 384 | -764 | 58 | 71 | 15 | 57 | -203 | 68 | 79 | 0.12 | 0.19 | 0.16 | 0.14 | 0.18 | + |
| Malathion | 114 | 88 | 135 | 87 | 62 | 72 | 90 | 4 | 56 | 79 | 0.16 | 0.11 | 1.73 | 0.19 | 0.18 | + |
| Mecoprop | 122 | 131 | NA | 105 | 94 | 47 | 50 | 0 | 42 | 73 | 2.00 | 2.05 | NA | 2.31 | 1.67 | - |
| Metalaxyl | 121 | 137 | -194 | 104 | 101 | 56 | 77 | -21 | 52 | 88 | 5.63 | 2.25 | 10.00 | 5.00 | 2.31 | + |
| Methiocarb | 112 | 111 | 492 | 92 | 81 | 73 | 83 | 6 | 64 | 82 | 0.35 | 0.29 | 1.41 | 0.29 | 0.27 | + |
| Methyl 3,4-dichlorophenylcarbamate (SWEP) | 110 | 91 | 42 | 146 | 147 | 184 | 150 | 5 | 78 | 91 | 0.20 | 0.22 | 3.21 | 0.48 | 0.38 | - |
| Methyl-benzotriazole | 103 | 130 | 64 | 107 | 165 | 64 | 72 | 5 | 47 | 69 | 0.05 | 0.05 | 0.69 | 0.10 | 0.04 | +/**-** |
| Molinate | 112 | 34 | -31 | 1117 | 977 | 804 | 1025 | 43 | 54 | 25 | 0.12 | 0.07 | 1.43 | 1.50 | 4.29 | + |
| Mono benzyl phthalate | 117 | 104 | 75 | 78 | 188 | 23 | 21 | 7 | 24 | 19 | 0.60 | 0.32 | 1.91 | 0.58 | 0.38 | - |
| Mono cyclohexyl phthalate | 111 | 136 | NA | 107 | 99 | 58 | 47 | 0 | 55 | 59 | 0.19 | 0.23 | NA | 0.17 | 0.19 | - |
| Mono isobutyl phthalate | 104 | 129 | 59 | 87 | 99 | 53 | 55 | 22 | 38 | 85 | 0.31 | 0.17 | 0.77 | 0.30 | 0.14 | - |
| Mono n-butyl phthalate | 117 | 129 | 19 | 73 | 139 | 28 | 53 | 6 | 37 | 77 | 0.40 | 0.16 | 0.99 | 0.37 | 0.14 | - |
| Mono(2-ethyl-5-hydroxyhexyl) Phthalate | 106 | 146 | -33 | 89 | 88 | 73 | 69 | 10 | 59 | 116 | 0.22 | 0.26 | 0.46 | 0.28 | 0.21 | - |
| Mono(2-ethyl-5-oxohexyl) Phthalate | 109 | 130 | NA | 84 | 104 | 65 | 96 | 0 | 61 | 105 | 0.15 | 0.11 | NA | 0.19 | 0.11 | - |
| Mono-2-ethylhexyl phthalate | 115 | 148 | 37 | 167 | 104 | 121 | 90 | 2 | 75 | 115 | 0.10 | 0.14 | 2.20 | 0.15 | 0.12 | - |
| Monooctyl Phthalate | 113 | 145 | 54 | 134 | 99 | 153 | 90 | 2 | 91 | 101 | 0.05 | 0.13 | 4.74 | 0.09 | 0.12 | - |
| Monopentyl Phthalate | 108 | 129 | 52 | 94 | 100 | 66 | 54 | 4 | 59 | 68 | 0.17 | 0.20 | 3.60 | 0.19 | 0.17 | - |
| N-acetyl sulfadiazine | 107 | 107 | 101 | 102 | 89 | 13 | 48 | 2 | 18 | 56 | 0.70 | 0.26 | 2.57 | 0.55 | 0.28 | + |
| N-acetyl sulfamethazine | 110 | 161 | 115 | 107 | 100 | 44 | 79 | 4 | 37 | 84 | 0.23 | 0.14 | 1.91 | 0.23 | 0.12 | **+**/- |
| N-acetyl sulfapyridine | 109 | 131 | 149 | 107 | 101 | 25 | 67 | 1 | 25 | 69 | 0.18 | 0.09 | 2.65 | 0.18 | 0.10 | **+**/- |
| Nalidixic acid | 110 | 141 | 25 | 89 | 77 | 41 | 58 | 1 | 40 | 82 | 0.06 | 0.04 | 2.20 | 0.04 | 0.03 | + |
| N-desmethyl venlafaxine | 107 | 146 | 67 | 104 | 100 | 29 | 52 | 1 | 28 | 60 | 0.06 | 0.03 | 1.14 | 0.06 | 0.03 | + |
| Nicotine | 118 | 127 | 100 | 173 | 114 | 17 | 41 | -3 | 8 | 45 | 0.48 | 0.18 | NA | 0.97 | 0.17 | + |
| O-desmethyl venlafaxine | 108 | 132 | 86 | 101 | 100 | 13 | 34 | 1 | 13 | 38 | 0.05 | 0.02 | 0.70 | 0.05 | 0.02 | **+**/- |
| Oxadiazon | 93 | 2 | 126 | 8 | 0 | 44 | 57 | 8 | 51 | 125 | 0.07 | 0.05 | 0.21 | 0.05 | 0.02 | + |
| Oxathiapiprolin | 120 | 152 | 399 | 11 | 25 | 49 | 73 | 2 | 66 | 132 | 15.00 | 15.00 | 5.00 | 15.00 | 15.00 | + |
| Oxolinic acid | 112 | 159 | NA | 87 | 42 | 29 | 42 | 0 | 25 | 105 | 0.67 | 0.42 | NA | 0.77 | 0.19 | + |
| Pilocarpine | 104 | 99 | NA | 119 | 100 | 9 | 33 | 0 | 11 | 38 | 0.22 | 0.08 | NA | 0.18 | 0.08 | + |
| Propanil | 104 | 145 | 246 | 93 | 91 | 59 | 57 | 2 | 34 | 57 | 0.09 | 0.10 | 0.84 | 0.15 | 0.12 | + |
| Sulfadimethoxine | 104 | 153 | 92 | 111 | 99 | 33 | 59 | 1 | 32 | 59 | 0.09 | 0.06 | 1.30 | 0.06 | 0.07 | **+**/- |
| Sulfamerazine | 102 | 71 | 111 | 104 | 101 | 11 | 46 | 1 | 18 | 46 | 0.32 | 0.06 | 2.05 | 0.13 | 0.05 | + |
| Sulfamethoxazole | 109 | 150 | 89 | 105 | 98 | 20 | 43 | 1 | 18 | 45 | 0.11 | 0.05 | 1.67 | 0.15 | 0.08 | + |
| Sulfamethoxypyridazine | 114 | 142 | 67 | 115 | 101 | 12 | 31 | 1 | 11 | 31 | 0.23 | 0.10 | 2.09 | 0.24 | 0.13 | **+**/- |
| Sulfaquinoxaline | 104 | 158 | 66 | 93 | 91 | 45 | 68 | 3 | 44 | 79 | 0.35 | 0.29 | 3.00 | 0.29 | 0.28 | + |
| Sulfathiazole | 107 | 379 | 110 | 103 | 89 | 16 | 15 | 1 | 3 | 16 | 0.27 | 0.25 | 2.90 | 1.67 | 0.24 | + |
| Terbumeton | 116 | 114 | 232 | 90 | 91 | 52 | 80 | 5 | 64 | 90 | 0.03 | 0.01 | 0.32 | 0.02 | 0.01 | + |
| Tonalide | 48 | 0 | 34 | 7 | -6 | 116 | 159 | 43 | 59 | 120 | 0.98 | 0.65 | 0.77 | 1.11 | 1.20 | + |
| Triclocarban | 88 | 66 | 15 | 2 | 5 | 113 | 77 | 6 | 79 | 101 | 0.04 | 0.06 | 0.80 | 0.06 | 0.04 | - |
| Tris(1-chloro-2-propyl) phosphate (TCPP) | 117 | 145 | -333 | 102 | 105 | 70 | 83 | -8 | 54 | 79 | 0.12 | 0.10 | 0.11 | 0.11 | 0.09 | + |
| Tris(2-chloroethyl) phosphate (TCEP) | 113 | 152 | 202 | 108 | 106 | 32 | 49 | 0 | 26 | 51 | 0.05 | 0.04 | 0.98 | 0.04 | 0.03 | + |
| Venlafaxine | 108 | 139 | 104 | 102 | 99 | 32 | 57 | 3 | 33 | 65 | 0.02 | 0.01 | 0.26 | 0.02 | 0.01 | + |
| Zoxamide | 107 | 81 | 295 | 60 | 30 | 47 | 61 | 6 | 52 | 94 | 0.30 | 0.23 | 1.48 | 0.26 | 0.16 | + |

^a^Matrix effect (ME%), ^b^Limit of detection (LOD), estimated solely for the purpose of comparing sample treatments.", ^c^Ionization mode (IM) positive (+), negative (-) or both (+/-).^d^Non-available (NA).

**SI – 6. Instrumental parameters optimization**

**Figure S3.** Injection volume optimization.


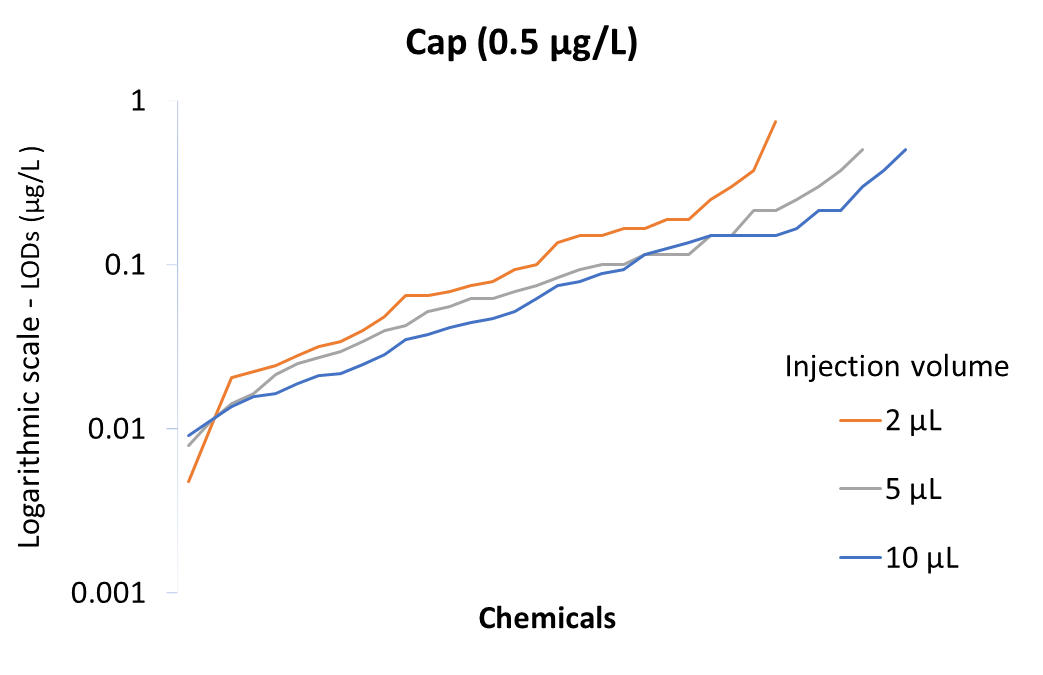


LODs (expressed in a logarithmic scale) for an urine sample spiked at 0.5 µg/L. Results for three different injection volumes.

**SI-7. Methods performance**

**Table S5**. Validation results for Cap protocol.

| Chemical name | Extraction efficiency | | | MF%^a^ | Reprod.^b^ | | LOD ^c^  (ng/mL) | LOQ ^d^  (ng/mL) | Linear range  (ng/mL) | R^2^ | IM^e^ |
| --- | --- | --- | --- | --- | --- | --- | --- | --- | --- | --- | --- |
|  | **2 ng/mL** | **10 ng/mL** | **50 ng/mL** |  | Intraday (n=9) | Interday (n=9) |  |  |  |  |  |
| 1,2,3,4-Tetrahidro-9H-pirido[3,4-B]Indole | <LOQ^f^ | 81±12 | 89±5 | 11 | 6 | 40 | 1.5 | 5 | 5 - 100 | 0.998 | + |
| 1h,1h,2h,2h-perfluorooctanesulfonic acid (6:2 FTS) | 268±9 | 159±14 | 136±6 | 116 | 3 | 16 | 0.06 | 0.2 | 0.2 - 100 | 0.999 | - |
| 2,2'-Dihydroxy-4-methoxybenzophenone | 74±16 | 49±13 | 57±11 | 83 | 9 | 8 | 0.15 | 0.5 | 0.5 - 100 | 0.999 | - |
| 2-amino-Benzothiazole | 76±5 | 76±12 | 86±6 | 24 | 4 | 15 | 0.03 | 0.1 | 0.1 - 100 | 0.998 | + |
| 2-Benzothiazolesulfonic acid | 154±10 | 97±12 | 106±7 | 8 | 7 | 5 | 0.15 | 0.5 | 0.5 - 100 | 0.998 | - |
| 2-Hydroxy-5-octanoylbenzoic acid | 139±14 | 123±12 | 121±7 | 133 | 3 | 8 | 0.06 | 0.2 | 0.2 - 50 | 0.999 | - |
| 3,4-Dihydroxybenzoic acid | <LOQ | 71±9 | 82±9 | 56 | 6 | 22 | 1.5 | 5 | 5 - 100 | 0.998 | - |
| 3,4-Dihydroxybenzoic Acid Methyl Ester | 73±6 | 79±9 | 80±7 | 61 | 8 | 19 | 0.15 | 0.5 | 0.5 - 100 | 1.000 | - |
| 4,4'-Dihydroxybenzophenone | 80±7 | 68±10 | 83±7 | 62 | 6 | 10 | 0.06 | 0.2 | 0.2 - 100 | 0.999 | - |
| 4-Aminoantipyrine | 62±11 | 76±13 | 98±7 | 25 | 2 | 10 | 0.3 | 1 | 1 - 100 | 0.999 | + |
| 4-Hydroxybenzoic acid-benzyl ester | 79±9 | 72±9 | 78±8 | 90 | 7 | 9 | 0.015 | 0.05 | 0.05 - 100 | 0.998 | - |
| 4-Hydroxybenzoic acid-isobutyl ester | 90±9 | 83±12 | 99±8 | 64 | 9 | 10 | 0.015 | 0.05 | 0.05 - 100 | 0.995 | - |
| 4-Hydroxybenzoic acid-isopropyl ester | 108±12 | 103±11 | 124±7 | 60 | 5 | 13 | 0.015 | 0.05 | 0.05 - 100 | 0.995 | - |
| 4-Hydroxybenzoic acid-n-butyl ester | 113±8 | 99±12 | 107±7 | 73 | 7 | 12 | 0.015 | 0.05 | 0.05 - 100 | 0.982 | - |
| 4-Hydroxybenzophenone | 114±5 | 84±7 | 93±3 | 15 | 8 | 10 | 0.015 | 0.05 | 0.05 - 100 | 0.997 | +/- |
| Alachlor | < LOQ | 100±14 | 114±7 | 68 | 8 | 11 | 1.5 | 5 | 5 - 100 | 0.996 | + |
| Atrazine | 132±15 | 100±13 | 111±6 | 33 | 6 | 19 | 0.15 | 0.5 | 0.5 - 100 | 1.000 | + |
| Benzophenone-1 | 91±8 | 72±7 | 81±7 | 19 | 5 | 9 | 0.15 | 0.5 | 0.5 - 100 | 0.999 | - |
| Benzophenone-2 | <LOQ | 111±7 | 88±13 | 21 | 8 | 17 | 0.6 | 2 | 1 - 100 | 0.999 | - |
| Benzophenone-3 | 79±9 | 72±9 | 78±8 | 90 | 8 | 9 | 0.015 | 0.05 | 0.05 - 100 | 0.998 | - |
| Benzophenone-4 | <LOQ | 119±19 | 98±6 | 77 | 13 | 21 | 1.5 | 5 | 5 - 100 | 0.993 | - |
| Bisphenol A | 120±11 | 81±10 | 83±7 | 51 | 6 | 7 | 0.15 | 0.5 | 0.5 - 100 | 1.000 | - |
| Bisphenol AF | 15±42 | 10±44 | 20±57 | 79 | 6 | 12 | 0.15 | 0.5 | 0.5 - 100 | 0.998 | - |
| Bisphenol AP | 22±56 | 16±32 | 31±53 | 66 | 7 | 13 | 0.15 | 0.5 | 0.5 - 100 | 0.999 | - |
| Bisphenol B | 86±3 | 61±14 | 72±8 | 56 | 6 | 8 | 0.15 | 0.5 | 0.5 - 100 | 0.998 | - |
| Bisphenol E | 80±14 | 68±17 | 82±7 | 56 | 6 | 8 | 0.15 | 0.5 | 0.5 - 100 | 1.000 | - |
| Bisphenol F | 64±18 | 54±20 | 58±4 | 74 | 10 | 9 | 1.5 | 5 | 5 - 100 | 1.000 | - |
| Bisphenol G | 2±46 | 1±109 | 6±77 | 48 | 13 | 26 | 0.06 | 0.2 | 0.2 - 100 | 0.993 | - |
| Bisphenol M | 4±91 | 6±25 | 8±59 | 45 | 15 | 26 | 0.06 | 0.2 | 0.2 - 100 | 0.992 | - |
| Bisphenol P | 4±91 | 6±24 | 8±59 | 45 | 15 | 27 | 0.06 | 0.2 | 0.2 - 100 | 0.990 | - |
| Bisphenol S | 89±14 | 71±8 | 79±6 | 15 | 5 | 9 | 0.15 | 0.5 | 0.5 - 100 | 0.999 | - |
| Bisphenol Z | 37±18 | 32±13 | 50±17 | 82 | 8 | 14 | 0.15 | 0.5 | 0.5 - 100 | 0.998 | - |
| Bromacil | 67±38 | 116±14 | 107±6 | 33 | 8 | 21 | 1.5 | 5 | 5 - 100 | 0.996 | + |
| Carbamazepine | 81±10 | 86±10 | 98±7 | 52 | 4 | 14 | 1.5 | 5 | 5 - 50 | 1.000 | + |
| Carbendazim | 67±2 | 67±12 | 77±6 | 44 | 2 | 10 | 0.03 | 0.1 | 0.1 - 100 | 1.000 | + |
| Chlorpyriphos | < LOQ | 3±12 | 3±42 | 92 | 9 | 23 | 1.5 | 5 | 5 - 50 | 0.986 | + |
| Clarithromycin | <LOQ | 118±40 | 172±16 | 12 | 41 | 26 | 3 | 10 | 10 - 100 | 0.999 | - |
| Dimethomorph | < LOQ | 75±5 | 76±7 | 78 | 9 | 21 | 1.5 | 5 | 5 - 100 | 1.000 | + |
| Dimethyl-benzotriazole | 83±7 | 69±7 | 81±6 | 52 | 6 | 12 | 0.015 | 0.05 | 0.05 - 100 | 0.995 | +/- |
| Diuron | 93±8 | 77±11 | 89±4 | 48 | 6 | 21 | 0.15 | 0.5 | 0.5 - 100 | 0.999 | +/- |
| Di-β,β'-Chloroethylphosphoric acid | 134±21 | 110±9 | 100±6 | 46 | 4 | 13 | 0.3 | 1 | 1 - 100 | 0.999 | - |
| Enrofloxacin | < LOQ | 42±53 | 55±9 | 113 | 6 | 9 | 1.5 | 5 | 5 - 100 | 0.998 | + |
| Enzacamene | 28±7 | 5±12 | 2±75 | 43 | 7 | 21 | 0.03 | 0.1 | 0.1 - 50 | 0.983 | + |
| Ethyl 3,4-Dihydroxybenzoate | 93±22 | 73±9 | 83±8 | 62 | 5 | 15 | 0.15 | 0.5 | 0.5 - 100 | 0.997 | - |
| Ethyl paraben | < LOQ | < LOQ | 142±8 | 76 | 19 | 16 | 3 | 10 | 10 - 100 | 0.998 | + |
| Flumequine | 85±7 | 81±10 | 98±6 | 49 | 3 | 15 | 0.015 | 0.05 | 0.05 - 100 | 1.000 | + |
| Gemfibrozil | <LOQ | 92±11 | 113±8 | 85 | 15 | 17 | 1.5 | 5 | 5 - 100 | 0.997 | - |
| Hexadecylamine | 37±47 | 30±29 | 57±26 | 50 | 2 | 8 | 0.015 | 0.05 | 0.05 - 100 | 1.000 | + |
| Lauryl diethanolamide | <LOQ | 89±6 | 65±10 | 68 | 6 | 54 | 0.6 | 2 | 1 - 100 | 0.999 | + |
| Malathion | 74±8 | 65±11 | 78±9 | 56 | 2 | 15 | 0.15 | 0.5 | 0.5 - 100 | 0.997 | + |
| Mecoprop | <LOQ | 92±14 | 96±9 | 42 | 12 | 12 | 1.5 | 5 | 5 - 100 | 0.999 | - |
| Metalaxyl | <LOQ | 97±11 | 102±8 | 52 | 4 | 12 | 1.5 | 5 | 5 - 100 | 1.000 | + |
| Methiocarb | 90±3 | 76±10 | 86±7 | 64 | 3 | 16 | 0.15 | 0.5 | 0.5 - 100 | 0.999 | + |
| Methyl 3,4-dichlorophenylcarbamate (SWEP) | 98±15 | 85±12 | 103±9 | 78 | 7 | 12 | 0.15 | 0.5 | 0.5 - 100 | 0.997 | - |
| Methyl-benzotriazole | 93±5 | 84±10 | 97±6 | 47 | 5 | 14 | 0.015 | 0.05 | 0.05 - 100 | 0.999 | +/- |
| Molinate | 1156±27 | 1205±17 | 1723±7 | 54 | 6 | 23 | 0.3 | 1 | 1 - 100 | 0.997 | + |
| Mono benzyl phthalate | 66±13 | 66±12 | 88±7 | 24 | 4 | 19 | 0.3 | 1 | 1 - 100 | 0.996 | - |
| Mono cyclohexyl phthalate | 412±67 | 120±51 | 103±5 | 55 | 5 | 8 | 0.15 | 0.5 | 0.5 - 100 | 1.000 | - |
| Mono isobutyl phthalate | 728±4 | 214±4 | 110±6 | 38 | 8 | 18 | 0.15 | 0.5 | 0.5 - 100 | 1.000 | - |
| Mono n-butyl phthalate | 291±7 | 117±14 | 100±5 | 37 | 8 | 11 | 0.06 | 0.2 | 0.2 - 100 | 1.000 | - |
| Mono(2-ethyl-5-hydroxyhexyl) Phthalate | 275±10 | 121±10 | 109±7 | 59 | 5 | 9 | 0.3 | 1 | 1 - 100 | 1.000 | - |
| Mono(2-ethyl-5-oxohexyl) Phthalate | 131±10 | 92±11 | 94±7 | 61 | 2 | 8 | 0.06 | 0.2 | 0.2 - 100 | 1.000 | - |
| Mono-2-ethylhexyl phthalate | 379±27 | 192±13 | 164±9 | 75 | 5 | 23 | 0.06 | 0.2 | 0.2 - 100 | 0.999 | - |
| Monooctyl Phthalate | 140±11 | 133±9 | 141±9 | 91 | 3 | 19 | 0.06 | 0.2 | 0.2 - 100 | 1.000 | - |
| Monopentyl Phthalate | 96±10 | 87±13 | 99±7 | 59 | 4 | 10 | 0.06 | 0.2 | 0.2 - 100 | 0.999 | - |
| N-acetyl sulfadiazine | <LOQ | 109±8 | 111±8 | 18 | 11 | 21 | 1.5 | 5 | 5 - 100 | 0.999 | + |
| N-acetyl sulfamethazine | <LOQ | 91±9 | 105±6 | 37 | 5 | 13 | 1.5 | 5 | 5 - 100 | 1.000 | +/- |
| N-acetyl sulfapyridine | 121±8 | 90±12 | 104±6 | 25 | 5 | 15 | 0.15 | 0.5 | 0.5 - 100 | 0.999 | +/- |
| Nalidixic acid | 86±5 | 80±9 | 98±6 | 40 | 2 | 13 | 0.015 | 0.05 | 0.05 - 100 | 1.000 | + |
| N-desmethyl venlafaxine | 94±5 | 92±9 | 105±6 | 28 | 3 | 10 | 0.015 | 0.05 | 0.05 - 100 | 1.000 | + |
| Nicotine | <LOQ | 123±11 | 141±6 | 8 | 7 | 6 | 1.5 | 5 | 5 - 100 | 0.996 | + |
| O-desmethyl venlafaxine | 82±6 | 80±9 | 100±7 | 13 | 3 | 15 | 0.06 | 0.2 | 0.2 - 100 | 0.999 | +/- |
| Oxadiazon | 6±68 | 4±36 | 8±60 | 51 | 8 | 27 | 0.06 | 0.2 | 0.2 - 100 | 0.996 | + |
| Oxathiapiprolin | <LOQ | 6±105 | 24±50 | 66 | 15 | 27 | 1.5 | 5 | 5 - 100 | 0.998 | + |
| Oxolinic acid | <LOQ | 91±11 | 88±5 | 25 | 2 | 17 | 1.5 | 5 | 5 - 100 | 0.999 | + |
| Pilocarpine | 262±4 | 126±10 | 115±6 | 11 | 32 | 14 | 0.15 | 0.5 | 0.5 - 100 | 1.000 | + |
| Propanil | 95±7 | 85±14 | 96±6 | 34 | 10 | 26 | 0.15 | 0.5 | 0.5 - 100 | 1.000 | + |
| Sulfadimethoxine | 100±2 | 95±9 | 112±7 | 32 | 21 | 18 | 0.15 | 0.5 | 0.5 - 100 | 0.999 | +/- |
| Sulfamerazine | 109±11 | 87±7 | 101±6 | 18 | 9 | 12 | 0.06 | 0.2 | 0.2 - 100 | 0.999 | + |
| Sulfamethoxazole | 103±4 | 92±8 | 110±7 | 18 | 4 | 16 | 0.15 | 0.5 | 0.5 - 100 | 0.999 | + |
| Sulfamethoxypyridazine | 97±5 | 92±10 | 108±5 | 11 | 7 | 12 | 0.15 | 0.5 | 0.5 - 100 | 0.999 | +/- |
| Sulfaquinoxaline | 108±11 | 84±8 | 95±4 | 44 | 4 | 10 | 0.15 | 0.5 | 0.5 - 100 | 0.999 | + |
| Sulfathiazole | <LOQ | 118±12 | 124±7 | 3 | 6 | 17 | 1.5 | 5 | 5 - 100 | 0.999 | + |
| Terbumeton | 77±5 | 78±12 | 98±7 | 64 | 3 | 9 | 0.015 | 0.05 | 0.05 - 100 | 1.000 | + |
| Tonalide | 49±17 | 8±23 | 3±47 | 59 | 10 | 32 | 0.15 | 0.5 | 0.5 - 50 | 0.983 | + |
| Triclocarban | 3±74 | 2±47 | 11±81 | 79 | 6 | 10 | 0.06 | 0.2 | 0.2 - 100 | 0.999 | - |
| Tris(1-chloro-2-propyl) phosphate (TCPP) | 248±6 | 121±11 | 106±5 | 54 | 6 | 13 | 0.015 | 0.05 | 0.05 - 100 | 1.000 | + |
| Tris(2-chloroethyl) phosphate (TCEP) | 100±6 | 96±12 | 108±5 | 26 | 3 | 14 | 0.03 | 0.1 | 0.1 - 100 | 0.999 | + |
| Venlafaxine | 92±4 | 92±8 | 105±6 | 33 | 2 | 7 | 0.015 | 0.05 | 0.05 - 100 | 1.000 | + |
| Zoxamide | 64±28 | 53±16 | 65±10 | 52 | 8 | 19 | 0.06 | 0.2 | 0.2 - 100 | 1.000 | + |

^a^Matrix factor (MF%), ^b^Reproducibility intra-day (n=9). ^c^Limit of detection (LOD). ^d^Limit of quantification (LOQ). ^e^Ionization mode (IM) in ESI (positive=+ and/or negative=-). ^f^Under the LOD (< LOD).

**SI-8. Deconjugation in HRMS-based strategies**

Cap-Glu protocol has been validated in the same way than Cap protocol, as explained in section **2.5.2 Methods validation parameters.** The extraction efficiency has been done at a 10 ng/mL fortification level.

**Table S6**. Validation results for Cap-Glu protocol.

| Chemical name | Extraction efficiency  (10 ng/mL) | MF%^a^ | LOD^b^  (ng/mL) | LOQ^c^  (ng/mL) | Linear range  (ng/mL) | R^2^ | IM^d^ |
| --- | --- | --- | --- | --- | --- | --- | --- |
| 1,2,3,4-Tetrahidro-9H-pirido[3,4-B]Indole | 96±13 | 17 | 1.5 | 5 | 5 - 100 | 0.989 | + |
| 1h,1h,2h,2h-perfluorooctanesulfonic acid (6:2 FTS) | 72±38 | 147 | 0.015 | 0.05 | 0.05 - 100 | 0.999 | - |
| 2,2'-Dihydroxy-4-methoxybenzophenone | 33±35 | 104 | 0.03 | 0.1 | 0.1 - 100 | 0.992 | - |
| 2-amino-Benzothiazole | 99±9 | 49 | 0.015 | 0.05 | 0.05 - 100 | 0.997 | + |
| 2-Benzothiazolesulfonic acid | 100±4 | 18 | 0.15 | 0.5 | 0.5 - 100 | 0.988 | - |
| 2-Hydroxy-5-octanoylbenzoic acid | 156±27 | 149 | 0.03 | 0.1 | 0.1 - 100 | 0.972 | - |
| 3,4-Dihydroxybenzoic acid | 108±20 | 86 | 1.5 | 5 | 5 - 100 | 0.997 | - |
| 3,4-Dihydroxybenzoic Acid Methyl Ester | 121±13 | 84 | 0.3 | 1 | 1 - 100 | 0.997 | - |
| 4,4'-Dihydroxybenzophenone | 97±24 | 104 | 0.3 | 1 | 1 - 100 | 0.978 | - |
| 4-Aminoantipyrine | 85±13 | 52 | 0.15 | 0.5 | 0.5 - 100 | 0.999 | + |
| 4-Hydroxybenzoic acid-benzyl ester | 90±19 | 127 | 0.015 | 0.05 | 0.05 - 100 | 0.998 | - |
| 4-Hydroxybenzoic acid-isobutyl ester | 113±24 | 92 | 0.015 | 0.05 | 0.05 - 100 | 1.000 | - |
| 4-Hydroxybenzoic acid-isopropyl ester | 164±19 | 83 | 0.015 | 0.05 | 0.05 - 100 | 1.000 | - |
| 4-Hydroxybenzoic acid-n-butyl ester | 146±21 | 95 | 0.015 | 0.05 | 0.05 - 100 | 0.991 | - |
| 4-Hydroxybenzophenone | 94±11 | 29 | 0.015 | 0.05 | 0.05 - 100 | 0.999 | +/- |
| Alachlor | 70±29 | 48 | 1.5 | 5 | 5 - 100 | 0.999 | + |
| Atrazine | 113±12 | 51 | 0.3 | 1 | 1 - 100 | 0.993 | + |
| Benzophenone-1 | 130±3 | 38 | 0.015 | 0.05 | 0.05 - 100 | 0.992 | - |
| Benzophenone-2 | 154±11 | 42 | 0.15 | 0.5 | 0.5 - 100 | 0.941 | - |
| Benzophenone-3 | 90±19 | 127 | 0.015 | 0.05 | 0.05 - 100 | 0.998 | - |
| Benzophenone-4 | 186±13 | 98 | 1.5 | 5 | 5 - 100 | 0.990 | - |
| Bisphenol A | 119±21 | 82 | 0.3 | 1 | 1 - 100 | 0.999 | - |
| Bisphenol AF | 29±19 | 124 | 0.015 | 0.05 | 0.05 - 100 | 0.998 | - |
| Bisphenol AP | 39±7 | 100 | 0.15 | 0.5 | 0.5 - 100 | 0.998 | - |
| Bisphenol B | 76±19 | 95 | 0.06 | 0.2 | 0.2 - 100 | 0.991 | - |
| Bisphenol E | 111±17 | 76 | 0.3 | 1 | 1 - 100 | 0.999 | - |
| Bisphenol F | 46±35 | 97 | 0.3 | 1 | 1 - 100 | 0.988 | - |
| Bisphenol G | 3±39 | 118 | 0.06 | 0.2 | 0.2 - 100 | 0.999 | - |
| Bisphenol M | 3±41 | 152 | 0.015 | 0.05 | 0.05 - 100 | 0.999 | - |
| Bisphenol P | 3±38 | 151 | 0.015 | 0.05 | 0.05 - 100 | 0.999 | - |
| Bisphenol S | 116±4 | 32 | 0.15 | 0.5 | 0.5 - 100 | 0.997 | - |
| Bisphenol Z | 42±14 | 126 | 0.06 | 0.2 | 0.2 - 100 | 0.995 | - |
| Bromacil | 134±10 | 56 | 1.5 | 5 | 5 - 100 | 0.994 | + |
| Carbamazepine | 102±15 | 81 | 0.15 | 0.5 | 0.5 - 50 | 1.000 | + |
| Carbendazim | 124±10 | 72 | 0.015 | 0.05 | 0.05 - 100 | 0.999 | + |
| Chlorpyriphos | NA | 180 | 0.15 | 0.5 | 0.5 - 100 | 0.991 | + |
| Clarithromycin | 60±53 | 47 | 1.5 | 5 | 5 - 100 | 0.995 | - |
| Di-β,β'-Chloroethylphosphoric acid | 121±11 | 150 | 0.03 | 0.1 | 0.1 - 100 | 0.993 | - |
| Dimethomorph | 74±10 | 90 | 0.3 | 1 | 1 - 100 | 0.991 | + |
| Dimethyl-benzotriazole | 139±19 | 64 | 0.03 | 0.1 | 0.1 - 100 | 0.991 | +/- |
| Diuron | 91±12 | 69 | 0.15 | 0.5 | 0.5 - 100 | 0.991 | +/- |
| Enrofloxacin | 40±5 | 584 | 0.15 | 0.5 | 0.5 - 100 | 0.991 | + |
| Enzacamene | 2±62 | 93 | 0.015 | 0.05 | 0.05 - 100 | 1.000 | + |
| Ethyl 3,4-Dihydroxybenzoate | 114±18 | 101 | 0.3 | 1 | 1 - 100 | 0.998 | - |
| Ethyl paraben | < LOD^e^ | 64 | 3 | 10 | 10 - 100 | 0.994 | + |
| Flumequine | 67±13 | 99 | 0.015 | 0.05 | 0.05 - 50 | 1.000 | + |
| Gemfibrozil | 104±28 | 103 | 0.15 | 0.5 | 0.5 - 100 | 0.991 | - |
| Hexadecylamine | 78±8 | 79 | 0.03 | 0.1 | 0.1 - 100 | 0.996 | + |
| Lauryl diethanolamide | 47±17 | 79 | 0.15 | 0.5 | 0.5 - 100 | 0.983 | + |
| Malathion | 43±16 | 79 | 0.15 | 0.5 | 0.5 - 100 | 0.990 | + |
| Mecoprop | 104±32 | 73 | 1.5 | 5 | 5 - 100 | 0.995 | - |
| Metalaxyl | 107±14 | 88 | 0.3 | 1 | 1 - 100 | 0.994 | + |
| Methiocarb | 64±15 | 82 | 0.15 | 0.5 | 0.5 - 100 | 0.988 | + |
| Methyl 3,4-dichlorophenylcarbamate (SWEP) | 98±23 | 91 | 0.3 | 1 | 1 - 100 | 0.994 | - |
| Methyl-benzotriazole | 158±13 | 69 | 0.003 | 0.01 | 0.01 - 100 | 0.991 | - |
| Molinate | 232±25 | 25 | 3 | 10 | 10 - 100 | 0.988 | + |
| Mono benzyl phthalate | 93±26 | 19 | 0.3 | 1 | 1 - 100 | 0.951 | - |
| Mono cyclohexyl phthalate | 106±16 | 59 | 0.015 | 0.05 | 0.05 - 100 | 0.994 | - |
| Mono isobutyl phthalate | 184±25 | 85 | 0.3 | 1 | 1 - 100 | 0.993 | - |
| Mono n-butyl phthalate | 187±24 | 77 | 0.3 | 1 | 1 - 100 | 0.992 | - |
| Mono(2-ethyl-5-hydroxyhexyl) Phthalate | 105±23 | 116 | 0.3 | 1 | 1 - 100 | 0.997 | - |
| Mono(2-ethyl-5-oxohexyl) Phthalate | 126±12 | 105 | 0.015 | 0.05 | 0.05 - 100 | 0.999 | - |
| Mono-2-ethylhexyl phthalate | 115±37 | 115 | 0.015 | 0.05 | 0.05 - 100 | 0.995 | - |
| Monooctyl Phthalate | 95±33 | 101 | 0.03 | 0.1 | 0.1 - 100 | 0.993 | - |
| Monopentyl Phthalate | 104±20 | 68 | 0.06 | 0.2 | 0.2 - 100 | 0.995 | - |
| N-acetyl sulfadiazine | 105±2 | 56 | 0.03 | 0.1 | 0.1 - 100 | 0.999 | + |
| N-acetyl sulfamethazine | 115±11 | 84 | 0.3 | 1 | 1 - 100 | 0.998 | +/- |
| N-acetyl sulfapyridine | 108±4 | 69 | 0.06 | 0.2 | 0.2 - 100 | 0.998 | +/- |
| Nalidixic acid | 91±11 | 82 | 0.03 | 0.1 | 0.1 - 100 | 0.990 | + |
| N-desmethyl venlafaxine | 118±10 | 60 | 0.015 | 0.05 | 0.05 - 100 | 0.992 | + |
| Nicotine | 106±9 | 45 | 0.15 | 0.5 | 0.5 - 100 | 0.992 | + |
| O-desmethyl venlafaxine | 108±5 | 38 | 0.015 | 0.05 | 0.05 - 100 | 0.996 | +/- |
| Oxadiazon | 0.3±65 | 125 | 0.015 | 0.05 | 0.05 - 50 | 0.995 | + |
| Oxathiapiprolin | 18±30 | 132 | 1.5 | 5 | 5 - 100 | 0.996 | + |
| Oxolinic acid | 48±4 | 105 | 0.15 | 0.5 | 0.5 - 50 | 0.998 | + |
| Pilocarpine | 108±32 | 38 | 0.3 | 1 | 1 - 100 | 0.990 | + |
| Propanil | 80±10 | 57 | 0.03 | 0.1 | 0.1 - 100 | 0.994 | + |
| Sulfadimethoxine | 104±12 | 59 | 0.15 | 0.5 | 0.5 - 100 | 0.997 | +/- |
| Sulfamerazine | 108±4 | 46 | 0.15 | 0.5 | 0.5 - 100 | 0.996 | + |
| Sulfamethoxazole | 104±6 | 45 | 0.03 | 0.1 | 0.1 - 100 | 0.995 | + |
| Sulfamethoxypyridazine | 109±2 | 31 | 0.06 | 0.2 | 0.2 - 100 | 0.999 | +/- |
| Sulfaquinoxaline | 120±17 | 79 | 0.15 | 0.5 | 0.5 - 100 | 0.992 | + |
| Sulfathiazole | 108±7 | 16 | 0.3 | 1 | 1 - 100 | 0.997 | + |
| Terbumeton | 92±16 | 90 | 0.015 | 0.05 | 0.05 - 100 | 0.999 | + |
| Tonalide | 2±144 | 120 | 0.015 | 0.05 | 0.05 - 100 | 0.998 | + |
| Triclocarban | 4±24 | 101 | 0.15 | 0.5 | 0.5 - 100 | 0.999 | - |
| Tris(1-chloro-2-propyl) phosphate (TCPP) | 97±18 | 79 | 0.015 | 0.05 | 0.05 - 100 | 0.995 | + |
| Tris(2-chloroethyl) phosphate (TCEP) | 105±9 | 51 | 0.015 | 0.05 | 0.05 - 100 | 0.998 | + |
| Venlafaxine | 127±11 | 65 | 0.015 | 0.05 | 0.05 - 100 | 0.986 | + |
| Zoxamide | 28±22 | 94 | 0.15 | 0.5 | 0.5 - 100 | 0.996 | + |

^a^Matrix factor (MF%), ^b^Limit of detection (LOD). ^c^Limit of quantification LOQ). ^d^Ionization mode (IM) in ESI (positive=+ and/or negative=-). ^e^Under the limit of detection (< LOD).

**Figure S.4.** Variations in Fold change across the samples


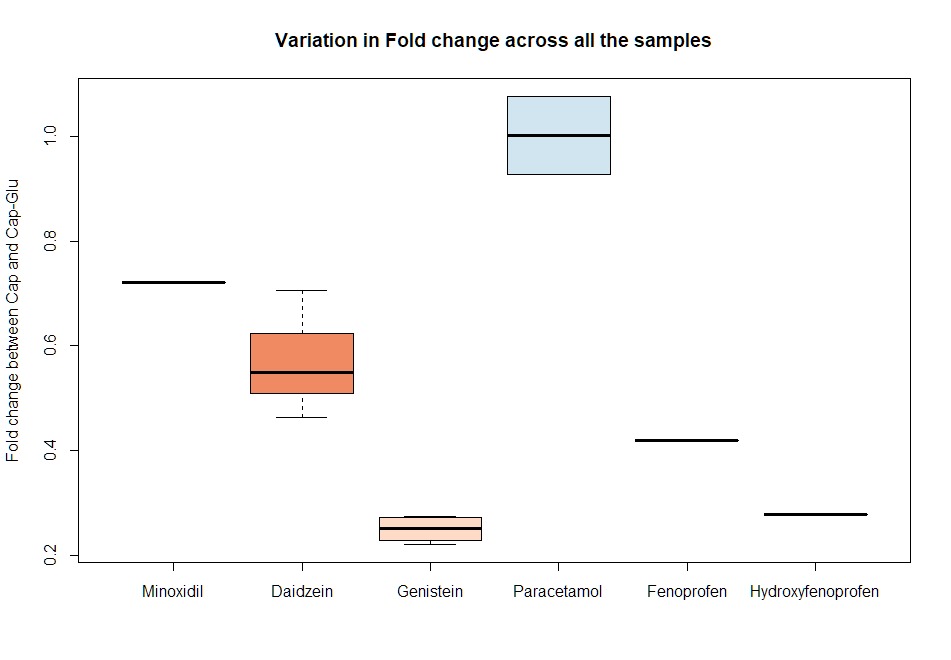

Supplement: Supplementary file 1 — Supplementary file1 (DOCX 476 KB) [file 216_2023_4998_MOESM1_ESM.docx]
